# Advancements in DNA‐Driven Precision Modulation of Cell Surface Receptor for Programmable Cellular Functions

**DOI:** 10.1002/advs.202505073

**Published:** 2025-06-30

**Authors:** Hexin Nan, Ming Cai, Yiyu Wang, Hong‐Hui Wang, Zhou Nie

**Affiliations:** ^1^ State Key Laboratory of Chemo and Biosensing College of Chemistry and Chemical Engineering College of Biology Hunan Provincial Key Laboratory of Biomacromolecular Chemical Biology Hunan University Changsha 410082 P. R. China

**Keywords:** cellular functions, DNA nanotechnology, dynamic DNA reactions, functional nucleic acids, synthetic biology

## Abstract

Precise modulation of receptor‐mediated signaling is essential for understanding cellular communication and developing targeted therapeutics. Receptor engineering strategies focus on enhancing specificity, manipulating allosteric effects, and controlling receptor clustering. This review comprehensively summarizes recent advances in DNA‐based strategies as versatile platforms for receptor engineering, encompassing both genetic and non‐genetic approaches. Genetic approaches leverage DNA's protein‐coding capability to reprogram receptor function through techniques like domain fusion and site‐directed mutagenesis. Complementarily, non‐genetic strategies exploit the structural and functional properties of DNA to achieve multidimensional control over receptor functionalities. Specifically, functional nucleic acids (FNAs) confer novel and customizable molecular recognition responsiveness, while DNA nanostructures, such as DNA origami, provide nanoscale spatial precision for regulating receptor valency and oligomerization. Furthermore, programmable dynamic DNA reactions facilitate the development of nanodevices responsive to diverse stimuli, including proteins, small molecules, ions, light, and mechanical forces. Notably, emerging DNA‐based logic circuits and nanorobots offer programmable and autonomous control over receptor signaling. Looking forward, integrating genetic and non‐genetic DNA engineering strategies holds significant promise at the interface of synthetic biology and DNA nanotechnology, driving the development of next‐generation intelligent cellular systems for precise medicine.

## Graphical abstract

DNA‐Based Genetic and Non‐Genetic Tools for Receptor Engineering. This review highlights recent advances in DNA‐based strategies for receptor engineering, including genetic approaches like domain fusion and site‐directed mutagenesis, as well as non‐genetic methods using functional nucleic acids, DNA nanostructures, and dynamic DNA reactions. These tools allow precise modulation of receptor functions to enable programmable cellular behaviors.

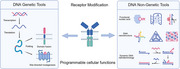

## 1 Introduction

Living cells, as the fundamental units of life, constantly encounter diverse environmental stimuli. To maintain homeostasisand drive adaptive evolution, cells must accurately sense, interpret, and respond to these signals.^[^ ^1^ ^]^ This capability is primarily mediated by cell surface receptors, which transduce extracellular cues into intracellular responses, orchestrating critical biological processes such as proliferation, differentiation, immune activation, and tissue repair.^[^ ^2^ ^]^ However, dysregulated receptor signaling arising from aberrant receptor expression, structural mutations, or intracellular pathway hyperactivation is implicated in various pathologies such as cancer, diabetes, and tissue injury.^[^ ^3^ ^]^ Therefore, the precise engineering of receptor functions is crucial for both elucidating cellular signaling mechanisms and developing novel therapeutic strategies.

Receptor functions are governed by two key aspects: ligand recognition specificity and activation through conformational changes or oligomerization.^[^ ^4^ ^]^ The receptor specificity arises from molecular complementarity between receptors and their ligands. Ligand binding or mechanical stimuli can induce receptor conformational changes, leading to dimerization or oligomerization, which amplifies downstream signaling.^[^ ^5^ ^]^ Many receptor families—including receptor tyrosine kinases (RTKs), integrins, and T‐cell receptors (TCRs)—undergo a transition from freely diffusive monomers to higher‐order oligomers upon ligand engagement, thereby amplifying downstream signaling cascades.^[^ ^6^, ^7^ ^]^ In nature, dynamic receptor clustering is essential for accurate signal recognition and transduction, underscoring its importance in regulating cellular behavior.^[^ ^8^ ^]^ Up to date, significant efforts have focused not only on dissecting the biochemical and biophysical principles governing receptor activation and clustering, but also on developing molecular engineering strategies for artificial control over receptor responsiveness, organization, and dynamics. This necessitates the development of novel tools and molecular systems capable of manipulating receptor function with high precision, specificity, and programmability.

**Figure 1:**
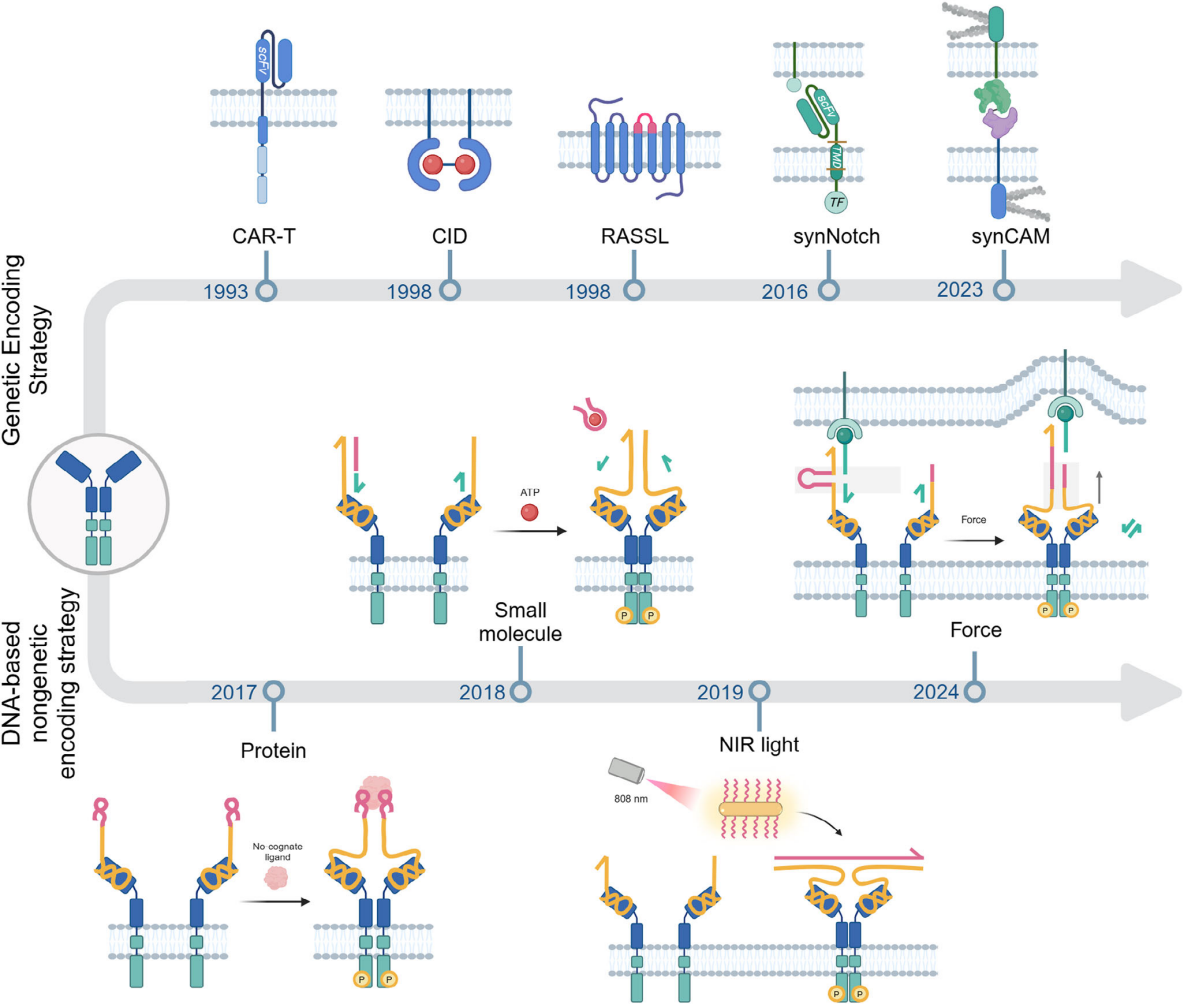
Historical Timeline of Key Milestones in DNA‐Based Genetic and Non‐Genetic Encoding Strategies for Receptor Engineering Over the Past Two Decades. Genetic encoding strategy (top): In 1993, Eshhar et al. engineered CAR‐T cells by fusing antibody‐derived antigen‐binding domains to T‐cell signaling modules, revolutionizing cancer immunotherapy.^[^ ^9^ ^]^ In 1998, Yang et al. introduced the CID strategy to spatiotemporally control receptor clustering via small molecules,^[^ ^10^ ^]^ while Coward et al. developed RASSLs (G protein‐coupled receptors responsive to synthetic ligands) to dissect signaling pathways without endogenous interference.^[^ ^11^ ^]^ In 2016, synNotch receptors emerged, enabling customizable sensing and response behaviors by modular replacement of extracellular and intracellular domains.^[^ ^12^ ^]^ By 2023, synCAM expanded cell engineering by programming cell‐cell interactions for multicellular system design.^[^ ^13^ ^]^ Non‐genetic encoding strategy (bottom): In 2017, Ueki et al. developed DNA aptamer‐mediated reprogramming of the interaction partner of receptor tyrosine kinases (DRIPaR), using DNA aptamers to reprogram growth factor receptor specificity via dynamic ligand‐receptor complexes.^[^ ^17^ ^]^ In 2018, Li et al. created DNA‐mediated chemically induced dimerization, a chemical‐responsive DNA nanodevice to trigger receptor dimerization with small‐molecule or ionic inputs.^[^ ^18^ ^]^ In 2019, Wang et al. pioneered NIR light‐activated DNA agonist, leveraging NIR light and gold nanorods for noninvasive in vivo control of DNA‐based agonists.^[^ ^20^ ^]^ In 2024, Yang et al. engineered DNA‐functionalized artificial mechanoreceptors to reprogram non‐mechanosensitive receptors for *de novo* mechanotransduction, advancing synthetic mechanobiology.^[^ ^21^ ^]^ Figures were created with BioRender.com, with permission.

To address this challenge, the emergence of DNA molecules as fundamental building blocks for molecular engineering platforms, including both genetic and non‐genetic approaches, offers unprecedented opportunities for targeted receptor modulation. The synthetic engineering of cell‐surface receptors aims to rewire transmembrane signaling pathways, enabling responsiveness to customized inputs or altering downstream outputs. Landmark advancements in genetic engineering of synthetic receptors including chimeric antigen receptors (CARs) (1993),^[^ ^9^ ^]^ chemically induced dimerization (CID) (1998),^[^ ^10^ ^]^ and receptors activated solely by synthetic ligands (RASSLs) (1998),^[^ ^11^ ^]^ progressing to more recent innovations such as synthetic Notch (synNotch) (2016),^[^ ^12^ ^]^ and synthetic cell adhesion molecule (synCAM) (2023)^[^ ^13^ ^]^ (**Figure** **1**). These synthetic biology‐driven approaches have progressively enhanced the specificity and functional versatility of engineered receptors by rewiring their response specificity, enabling customized recognition of novel inputs and efficient modulation of downstream cellular signaling.^[^ ^14^ ^]^ Nevertheless, genetic receptor engineering faces inherent limitations, including potential disruptions to endogenous cellular processes, variability in receptor expression, and challenges in preserving the correct functionality of engineered proteins.^[^ ^15^ ^]^ Beyond these issues, engineered receptors may suffer from off‐target effects, crosstalk reactions, and in vivo applications affected by factors such as the immune system, tissue microenvironment, and metabolic conditions. Such constraints highlight the need for alternative, non‐genetic receptor remodeling strategies capable of rewiring receptor response specificity to regulate signaling outputs and cellular behaviors without genetic alterations.

In this context, leveraging the intrinsic non‐genetic properties of DNA molecules represents a paradigm shift, significantly broadening the toolkit for flexible and response‐specific receptor rewiring.^[^ ^16^ ^]^ Over the past decade, this non‐genetic approach has expanded significantly, progressing from protein‐responsiveness (2017)^[^ ^17^ ^]^ and small molecule‐controlled DNA nanodevices (2018)^[^ ^18^ ^]^ to ultraviolet (UV) light‐responsive activation (2018)^[^ ^19^ ^]^ and near‐infrared (NIR) light‐triggered receptor modulation in vivo (2019)^[^ ^20^ ^]^ (Figure 1). More recently, force‐activated receptors (2023) have enabled *de novo* reconfiguration of mechanotransduction to customized cellular responses, further broadening the scope of non‐genetic receptor engineering.^[^ ^21^ ^]^ Therefore, DNA molecules—leveraging both genetic and non‐genetic modalities—represent a powerful, integrated molecular toolbox, profoundly reshaping receptor signaling control and cellular behavior manipulation toward intelligent and precision‐driven therapeutic strategies.

In this review, we discuss recent advances in DNA‐based receptor engineering, highlighting the integration of genetic or non‐genetic strategies to enhance cellular communication and targeted therapeutic interventions. DNA's molecular versatility enables receptor modulation through two complementary approaches. Genetic strategies utilize the protein‐encoding capabilities of DNA sequence, employing domain fusion and site‐directed mutagenesis to precisely reconfigure receptor specificity, affinity, and signaling dynamics.^[^ ^22^ ^]^ This enables rational construction of synthetic receptors with defined activation profiles and functional outcomes. In contrast, non‐genetic strategies exploit the intrinsic properties of DNA molecules, including structural programmability, predictable Watson–Crick base pairing, recognition, and biocatalytic functions, and dynamic self‐assembly to construct nanoscale scaffolds and stimulus‐responsive regulatory modules.^[^ ^16^ ^]^ Key molecular components of DNA‐based non‐genetic modules include functional nucleic acids (FNAs), such as aptamers and DNAzymes, which provide specific molecular recognition and catalytic functions.^[^ ^23^ ^]^ The DNA nanostructure‐based scaffolds enable spatial organization of receptor clustering and valency control.^[^ ^24^ ^]^ Notably, dynamic DNA reactions for the construction of DNA logic circuits and nanorobots provide a programmable and intelligent platform to regulate receptor‐mediated cellular behaviors.^[^ ^25^ ^]^ Building upon these molecular components, we outline major receptor engineering paradigms developed over the past two decades. We first summarize genetic receptor engineering strategies, including CAR‐T, synNotch, and synCAM, which have revolutionized synthetic biology and immunotherapy. We then examine non‐genetic receptor modulation, focusing on bivalent and multivalent receptor agonist regulation, nanoscale receptor organization via DNA origami, and stimulus‐responsive DNA architectures for programmable cellular modulation. Notably, DNA logic circuits and nanorobots introduce higher‐order programmability, enabling highly controlled, logic‐circuit‐controlled receptor activation and advanced cell behavior programming. With the progress and convergence of synthetic biology and DNA nanotechnology, we envision that integrating genetic and non‐genetic DNA engineering will transform receptor manipulation, potentially redefining receptor engineering and opening new avenues for precision medicine.

## 2 DNA‐Based Molecular Tools for Receptor Engineering

DNA molecules have emerged as powerful molecular tools to engineer and modulate receptor function, employing both genetic and non‐genetic strategies. As a genetic‐encoding material, DNA enables receptor engineering through approaches such as domain fusion and site‐directed mutagenesis, allowing precise modification of receptor responsiveness and downstream intracellular signaling pathways. Alternatively, DNA can serve as a non‐genetic molecular platform, facilitating the modular functionalization of endogenous receptors, providing user‐defined responsiveness, controlled receptor oligomerization, and dynamic regulation of receptor‐mediated cellular behaviors. This section briefly summarizes these genetic and non‐genetic DNA‐based methodologies for receptor engineering.

### 2.1 DNA as Encoding Molecule to Construct Synthetic Receptors

**Figure 2:**
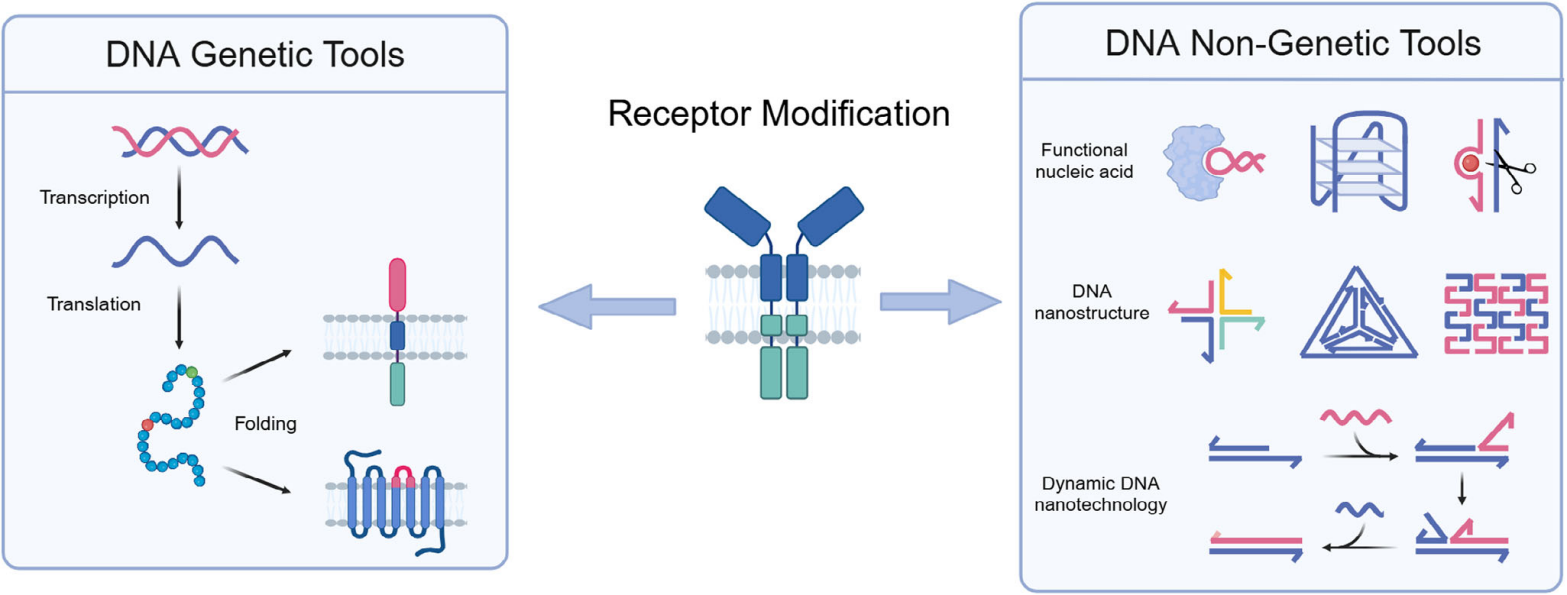
DNA‐based genetic and non‐genetic tools for receptor engineering. The scheme illustrates two complementary DNA‐driven strategies for receptor engineering. Genetic Tools (Left): These methods rely on DNA‐encoded transcription and translation to construct synthetic receptors with tunable properties. Strategies such as domain fusion and site‐directed mutagenesis enable precise receptor design and regulation, exemplified by CAR‐T engineering, where extracellular, transmembrane, and intracellular domains are strategically combined to achieve controlled expression, localization, and signal transduction. Non‐Genetic Tools (Right): These strategies exploit DNA's structural programmability to modulate receptor activity without altering genetic sequences. FNAs (e.g., aptamers, DNAzymes), DNA nanostructures (e.g., origami, tetrahedral frameworks), and dynamic DNA nanotechnology (e.g., strand displacement reactions) enable cue‐responsive customization and logic‐gated activation. Figures were created with BioRender.com, with permission.

DNA, as the fundamental carrier of genetic information, facilitates the synthetic receptor engineering through recombinant DNA techniques. By exploiting the central dogma, researchers can modularly fuse different receptor domains and introduce site‐directed mutations to fine‐tune structure‐function relationships (**Figure** **2**).^[^ ^26^ ^]^ Building upon fundamental insights into natural receptor functions, two major strategies have been developed for synthetic receptor construction, involving domain fusion and site‐directed mutagenesis.

#### 2.1.1 Domain Fusion for Custom Receptor Design

Cell‐surface receptors typically consist of extracellular sensing, transmembrane, and intracellular effector domains.^[^ ^27^ ^]^ Domain fusion leverages this modular architecture by recombining receptor domains to engineer synthetic receptors with novel functionalities.^[^ ^27^ ^]^ This approach is exemplified by CAR‐T cell therapy, where single‐chain fragment variant (scFv) domain swapping reprograms antigen specificity, and intracellular co‐stimulatory domains derived from the cell surface molecules (e.g., CD28, 4‐1BB) enhance T cell activation.^[^ ^28^ ^]^ Similarly, synNotch receptors exploit the mechanosensitive properties of the Notch core, incorporating customizable ligand‐binding domains (e.g., scFv) and transcription factors to regulate gene expression.^[^ ^29^ ^]^ To further optimize receptor signal transduction and specificity, researchers have refined transmembrane domain (TMD) and hinge region designs, particularly in synthetic intramembrane proteolysis receptors (SNIPR) and modular extracellular sensor architecture (MESA) systems. These optimizations reduce background activity, fine‐tune the force‐responsive threshold, and improve signal transduction efficiency.^[^ ^30^, ^31^ ^]^ By reconfiguration of the receptor architecture, the domain fusion approach enhances the specificity, affinity, and signal‐to‐noise ratios, optimizing receptor performance for therapeutic and research applications.

#### 2.1.2 Site‐Directed Mutagenesis for Functional Modulation

Given the sequence‐specific nature of receptor structure‐function relationships, precise modification of key amino acid residues via site‐directed mutagenesis enables targeted alteration of receptor function.^[^ ^32^ ^]^ This approach is particularly useful in G protein‐coupled receptor (GPCR) engineering, where targeted point mutations can modulate ligand specificity or signaling potency.^[^ ^32^ ^]^ Rationally designed GPCR variants, such as RASSLs, incorporate mutations that abolish native ligand responsiveness while permitting selective activation by synthetic drugs, achieving highly selective and orthogonal receptor control.^[^ ^22^, ^33^ ^]^ Additionally, directed evolution—iterative screening of receptor variants—further refines receptor function by enhancing binding affinity, signaling dynamics, and stability, all while preserving the receptor's core structural integrity.^[^ ^34^ ^]^ Thus, site‐directed mutagenesis provides a robust genetic strategy for achieving finely tuned receptor functions.

Together, these genetic engineering approaches highlight the remarkable versatility of DNA as an encoding molecule, enabling the adaptable design of synthetic receptors with tailored functionalities for diverse biomedical and therapeutic applications.

### 2.2 DNA as Nongenetic Molecular Platform to Engineer Receptors

Beyond its conventional role in genetic encoding, DNA possesses unique non‐genetic functionalities that serve as a transformative molecular platform for receptor modulation. Three primary DNA‐based regulatory modules, e.g., FNAs, DNA nanostructures, and dynamic DNA reactions, offer distinct yet synergistic capabilities to manipulate receptor functions, enabling sophisticated control over cellular behaviors (Figure 2). Specifically, DNA‐based receptor modulation toolkits involve three key modules categorized as: i) recognition modules (e.g., aptamers or DNA‐conjugated ligands (e.g., SNAP‐tag and Halo‐Tag)) that enable target binding with high specificity; ii) structural scaffolding modules (e.g., DNA origami) that facilitate spatial receptor organization with precise ligand presentation; and iii) logic execution modules (e.g., strand displacement reaction networks) that allow for responsive actions or complex signal processing.^[^ ^16^ ^]^

#### 2.2.1 FNAs

FNAs represent a powerful class of nucleic acid molecules, including aptamers and DNAzymes, that exhibit inherent molecular recognition and enzyme‐mimicking catalytic functions.^[^ ^35^ ^]^ Aptamers are single‐stranded DNA or RNA oligonucleotides that exhibit antibody‐like target‐recognizing specificity by forming high‐affinity binding pockets, providing molecular recognition tools in various biomedical applications. Selected through systematic evolution of ligands by exponential enrichment (SELEX) and its derivatives (e.g., Cell‐SELEX), aptamers bind small molecules, ions, proteins, and whole cells, with micromolar‐to‐picomolar affinities.^[^ ^36^, ^37^, ^38^ ^]^ Notably, numerous aptamers capable of high‐affinity targeting across diverse cell surface receptor classes have been extensively studied. These aptamer‐receptor pairs serve as molecular toolkits for DNA‐based receptor engineering via aptamer‐ binding‐mediated receptor manipulation, providing chemically programmable recognition interfaces and actuation domains that enable customized receptor modulation architectures. The rapid synthesis, batch‐to‐batch consistency, and structural adaptability of aptamers make them highly suitable for receptor binding and modulation in biosensing and precision medicine applications.^[^ ^39^ ^]^

DNAzymes, on the other hand, possess catalytic activities analogous to protein enzymes, enabling dynamic and responsive molecular functions.^[^ ^40^ ^]^ With programmable catalytic cores sensitive to specific cofactors (e.g., ions, metabolites), DNAzymes can be engineered to trigger dynamic responses, including molecular recognition and allosteric modulation of receptor functions.^[^ ^41^ ^]^ In receptor engineering applications, FNAs thus serve either as receptor‐binding modules through aptamer‐target interactions or as signal‐responsive elements via cofactor‐dependent DNAzyme activity.^[^ ^42^ ^]^ Furthermore, rational integration of FNAs with DNA nanostructures or dynamic DNA assemblies allows coupling molecular recognition events with sequential signal transduction processes, enabling controlled receptor clustering and sophisticated modulation of cellular signaling pathways.^[^ ^43^ ^]^

#### 2.2.2 DNA Nanostructures

The predictable Watson–Crick base‐pairing principle endows DNA molecules with unparalleled programmability, enabling accurate construction of well‐defined two‐ and 3D nanoscale architectures.^[^ ^44^ ^]^ Various DNA nanostructures have been designed with customizable shapes and sizes, including Seeman's four‐arm junctions, polyhedron DNA nanostructures, DNA origami, and single‐stranded DNA tiles (SST)‐based nanoassemblies.^[^ ^45^ ^]^ The significant advantage of DNA nanostructures is their precise spatial addressability, allowing accurate and organized ligand presentation to interact with receptors. This spatial organization effectively mimics physiological receptor oligomerization, a critical mechanism in natural cellular signaling pathways.^[^ ^46^ ^]^ By strategically inserting ligand‐functionalized DNA strands at defined stoichiometries and spatial arrangements, the assembled receptor clusters and downstream signaling efficiency can be finely optimized.^[^ ^47^ ^]^

#### 2.2.3 Dynamic DNA Reactions

Dynamic DNA reactions refer to the programmable interactions between DNA strands, enabling the construction of reconfigurable molecular reaction networks with highly controllable kinetic properties.^[^ ^48^ ^]^ These reactions are fundamental to dynamic DNA nanotechnology, where sequence‐specific hybridization and strand displacement mechanisms facilitate the development of molecular devices and circuits.^[^ ^49^ ^]^ Specifically, toehold‐mediated strand displacement (TMSD) reactions enable controlled strand exchange, with tunable kinetics determined by toehold sequence and length.^[^ ^50^ ^]^ This programmability underlies the design of DNA walkers, molecular motors, and logic gates.^[^ ^51^ ^]^ Additionally, signal amplification strategies such as hybridization chain reaction (HCR), catalytic hairpin assembly (CHA), and entropy‐driven DNA catalysis (EDC) enhance signal transduction efficiency through autonomous reaction networks.^[^ ^52^ ^]^ By integrating chemically modified aptamers, DNAzyme cleavage events, and dynamically structured FNAs, dynamic DNA reactions can be programmed to respond to environmental stimuli (e.g., proteins, small molecules, ions, light, nucleic acids, and forces). These programmable dynamic reactions facilitate the reconfiguration of highly specific target‐responsive signal transduction pathways.^[^ ^16^ ^]^ Importantly, DNA‐facilitated cascaded dynamic reactions enable multi‐layered strand displacement networks for precise receptor regulation.^[^ ^53^ ^]^ By constructing hierarchical reaction cascades, complex logic gates (e.g., AND, NIMPLY) have been implemented for conditional receptor activation with exceptional selectivity.^[^ ^54^ ^]^ Furthermore, these dynamic systems have been employed to power autonomous DNA nanorobots capable of sophisticated signal processing, ligand coupling, and controlled mechanical actions.^[^ ^55^ ^]^ Such advancements provide higher‐dimensional and intelligent strategies for receptor regulation, offering adaptive and programmable control mechanisms in synthetic biology and nanomedicine.

## 3 Genetically Engineered Synthetic Receptors

Advancements in genetic engineering have enabled the transformation of natural receptors into sophisticated synthetic counterparts, designed with customized sensing and response programs. By introducing engineered receptors, advanced synthetic biology strategies can link extracellular and intracellular cues to user‐defined cellular responses.^[^ ^56^, ^57^ ^]^ This section summarizes key developments in synthetic receptor engineering, highlighting how these platforms enable precise cellular control and broaden applications in immunotherapy, synthetic biology, and engineered cell therapies.

### 3.1 Synthetic Receptor Engineering

Inspired by natural receptor mechanisms, synthetic receptors are rationally designed to achieve customizable cellular control.^[^ ^58^ ^]^ These receptors integrate three key modular components: 1) Extracellular recognition domain, which is scFvs or designed proteins ensure high specificity and dynamic responsiveness. 2) TMD, which can be optimized to enhance signal transmission and membrane localization. 3) Intracellular effector domain, which incorporates co‐stimulatory domains and gene regulatory elements to translate extracellular recognition into precise functional outputs (e.g., targeted killing, gene editing). By modularly fusing these functional components via a domain fusion strategy, synthetic receptors expand the function of natural receptors and can evolve into a programmable platform for precise therapeutic applications and advanced cellular engineering.^[^ ^59^, ^60^ ^]^

#### 3.1.1 CARs

CARs exemplify modular synthetic receptor design, integrating antigen recognition, structural anchoring, and signal transduction components. Over five generations, CAR technology has evolved, with key improvements in intracellular signaling domains and cytokine/co‐stimulatory molecule incorporation to enhance therapeutic efficacy and persistence.^[^ ^61^ ^]^ One notable innovation in CAR engineering was introduced by Lim et al. in 2015, who developed a rapamycin‐inducible CAR switch, enabling precise, small‐molecule‐controlled CAR‐T activation.^[^ ^62^ ^]^ Beyond this, logic‐gated CAR circuits now integrate Boolean logic (AND/OR/NOT) functions, refining antigen specificity and reducing off‐target effects.^[^ ^63^ ^]^ These advancements demonstrate the increasing sophistication of programmable CAR platforms, expanding applications in cancer immunotherapy, autoimmune disease treatment, and precision medicine.

#### 3.1.2 SynNotch Receptors

Inspired by the mechanosensitive Notch receptor, synNotch receptor consists of an extracellular binding domain, a transmembrane regulatory domain, and an intracellular transcription factor module. Ligand binding triggers proteolytic cleavage, releasing transcription factors to regulate gene expression in a customizable manner.^[^ ^27^, ^61^ ^]^ Recent applications of synNotch receptors include tissue‐specific therapeutic strategies. For instance, engineered central nervous system (CNS)‐targeting T cells can express IL‐10 to suppress neuroinflammation or CARs to eliminate intracranial tumors without systemic toxicity.^[^ ^64^ ^]^ Moreover, the responsiveness of synNotch can be extended to intercellular forces. By integrating force‐sensitive protein modules, tension‐tunable synNotch receptors have been developed that respond to mechanical forces between cells, offering new tools for studying mechanotransduction.^[^ ^12^ ^]^

#### 3.1.3 SynCAMs

Natural intercellular interactions are mediated by various cell adhesion molecules, which are complex transmembrane proteins functioning as membrane‐bound ligand‐receptor interactions. These molecules can bind to adjacent cells or the extracellular matrix, inducing mechanical adhesion responses. Based on the natural cadherin family, Lim et al. have recently engineered synCAMs to provide customized input force‐output cell signaling pathways.^[^ ^13^ ^]^ These engineered adhesion molecules facilitate customized tissue assembly, organoid engineering, and mechanobiological studies, enhancing synthetic tissue construction and cellular morphology control.

#### 3.1.4 Programmable Antigen‐Gated GPCRs (PAGERs)

While CAR and synNotch receptors have been widely applied, their activation mechanisms—antigen‐induced clustering and mechanical force, respectively—limit the range of antigens and outputs. To address these constraints, Ting et al. developed programmable antigen‐gated G‐protein‐coupled engineered receptors.^[^ ^65^ ^]^ PAGERs utilize the domain fusion strategy to integrate nanobodies and auto‐inhibitory domains to the extracellular N‐terminus of GPCRs. When an antigen binds to the nanobody, it causes a conformational change that sterically occludes the auto‐inhibitory domain from binding to the GPCR, relieving the auto‐inhibition and allowing the receptor to be activated by a drug, thereby converting antigen binding into downstream GPCR signals. This platform expands the repertoire of synthetic receptors, providing new possibilities for programming cellular behavior with built‐in drug control mechanisms.

Collectively, synthetic receptors such as CARs, synNotch, synCAMs, and PAGERs serve as paradigmatic examples of synthetic biology‐driven receptor engineering. By integrating modular design principles with cutting‐edge genetic engineering strategies, these platforms enable highly customizable input‐output functions tailored to specific therapeutic or research needs.

### 3.2 Chemogenetic Approaches

Chemogenetics combines chemistry and genetics to engineer receptors responsive to synthetic ligands, enabling spatiotemporal cellular control while minimizing endogenous crosstalk. By developing artificial receptors that interact exclusively with synthetic molecules, these strategies offer a highly tunable and orthogonal control mechanism.

#### 3.2.1 Protein Labeling Technologies

Real‐time receptor tracking and direct modulation remain challenging, necessitating the development of engineered protein tags for receptor imaging and modulation.^[^ ^66^ ^]^ SNAP‐Tag, derived from O^6^‐alkylguanine‐DNA alkyltransferase (hAGT), forms irreversible covalent bonds with BG derivatives, enabling rapid, high‐sensitivity labeling and live‐cell imaging of receptor dynamics.^[^ ^67^, ^68^ ^]^ Similarly, Halo‐Tag, utilizing haloalkane dehalogenase, irreversibly binds to the functionalized substrates, making it ideal for long‐term receptor tracking and functional manipulation in complex biological systems.^[^ ^69^ ^]^ These complementary tagging systems provide a versatile toolkit for receptor visualization, regulation, and multiplexed control, enhancing applications in synthetic biology and receptor‐based therapies.

Recently, chemogenetic RTKs showcase the integration of ligand‐binding domains with photo‐switchable or chemically responsive elements. For instance, replacing RTK extracellular domains with metabotropic glutamate receptor 2 (mGluR2) ligand‐binding regions and attaching glutamate‐based photo‐switches generated optogenetic RTKs (LihIR and LihMET), enabling light‐controlled activation of insulin or MET signaling pathways.^[^ ^70^ ^]^ Additionally, Knaus et al. utilized the Halo‐Tag and SNAP‐Tag system to label bone morphogenetic protein (BMP)/transforming growth factor β (TGFβ) receptors, allowing specific fluorescence labeling and real‐time imaging of cell surface receptors and ligand‐receptor interactions, with receptor modifications further refining ligand specificity and signaling properties.^[^ ^71^ ^]^ These advancements highlight chemogenetics' potential in receptor modulation for regenerative medicine and synthetic signaling networks.

#### 3.2.2 Genetic Code Expansion (GCE) Technology

GCE introduces unnatural amino acids (UAAs) into proteins, expanding their functional diversity beyond natural amino acid constraints.^[^ ^72^ ^]^ UAAs enhance protein stability, catalytic activity, and post‐translational modification mimicry, enabling precise receptor engineering.^[^ ^73^ ^]^ For example, photocaged UAAs have enabled light‐dependent activation of key signaling proteins such as luciferase, kinases, and GTPase.^[^ ^74^ ^]^ Moreover, bioorthogonal UAAs facilitate covalent drug conjugation, exemplified by C−C chemokine receptor 5 (CCR5) engineered with noncanonical amino acids (ncAAs) for targeted GPCR modulation via bioorthogonal maraviroc analog conjugation.^[^ ^75^ ^]^

Together, chemogenetic strategies integrate protein tagging, optogenetics, and synthetic amino acid incorporation to achieve selective and highly specific receptor regulation. These approaches provide spatiotemporal precision, expanding receptor engineering capabilities in biomedical applications.

### 3.3 Advantages and Challenges of the Synthetic Receptor

The design and modification of synthetic cell‐surface receptors hold considerable promise across multiple fields, notably synthetic biology, biomedical research, and therapeutic development. Synthetic receptors confer several key advantages.

First, engineered receptors offer enhanced specificity and affinity, improving their capacity to recognize distinct molecules or biological signals. Such enhanced recognition is particularly beneficial for disease diagnostics and targeted therapies, exemplified by receptors engineered to detect tumor‐associated biomarkers for early cancer diagnosis. Second, receptor engineering facilitates optimized signal transduction. By rationally modifying receptor domains, researchers can fine‐tune signaling dynamics, increasing cellular sensitivity and responsiveness to external environmental cues—an essential consideration in synthetic biology applications. Third, synthetic receptors can integrate novel functionalities, including modules for intercellular communication or biosensing capabilities, allowing real‐time environmental monitoring or dynamic control of cellular interactions. Finally, chemogenetically engineered receptors serve as versatile tools to dissect specific biological processes or signaling pathways, providing valuable insights into cellular and physiological mechanisms.

Despite these significant advances, challenges persist in synthetic receptor design. These include design complexity, difficulties in robust and controlled receptor expression, potential limitations in achieving sufficient specificity, and implementation robustness in complex biological environments. Addressing these challenges is crucial for realizing the full potential of synthetic receptor strategies in translational applications.

## 4 DNA‐based Non‐Genetic Engineering of Receptor‐mediated Cell Regulation

**Figure 3:**
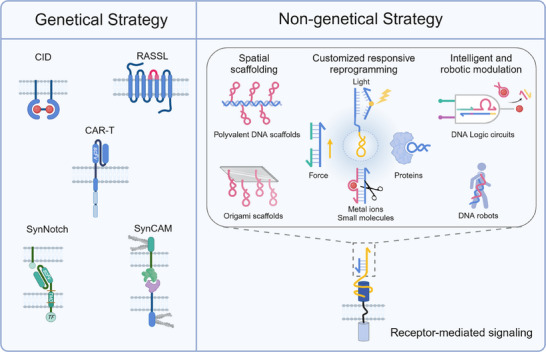
DNA‐based Genetic and Non‐Genetic Strategies for Receptor Modulation. The schematic illustrates two distinct approaches for engineering receptor function: genetically modified and non‐genetically modified strategies. Genetically Modified Receptors (Left): These methods employ DNA‐based transcriptional and translational engineering to construct synthetic receptors with programmable responses. Examples include CID, CAR‐T, receptor‐based synthetic biology platforms such as synNotch and synCAM, and engineered GPCRs like RASSLs, enabling controlled receptor activation and cellular reprogramming. Non‐Genetically Modified Receptors (Right): These strategies leverage DNA nanotechnology for receptor modulation without genetic alterations. Approaches include polyvalent DNA ligands, DNA origami scaffolds for spatial receptor organization, and programmable DNA‐based logic circuits that enable customized receptor reprogramming in response to diverse environmental cues (e.g., proteins, metal ions, mechanical forces, and light). DNA nanorobots further enable autonomous receptor‐mediated cellular functions, expanding receptor control beyond conventional genetic constraints. Figures were created with BioRender.com, with permission.

Compared to traditional genetic engineering methods for receptor modulation, DNA‐based non‐genetic strategies offer enhanced modularity, flexibility, and programmability for highly controlled receptor regulation. Leveraging the unique properties of FNAs, DNA nanostructures, and dynamic DNA reactions, these approaches enable receptor regulation through bivalent or polyvalent ligand assembly, spatially organized presentation, stimulus‐responsive dynamics, and logic‐gated activation (**Figure** **3**). These innovative DNA‐based non‐genetic tools significantly expand the capabilities for finely tuned receptor engineering for customized cellular functions.

### 4.1 Bivalent DNA‐based Receptor Agonists

**Figure 4:**
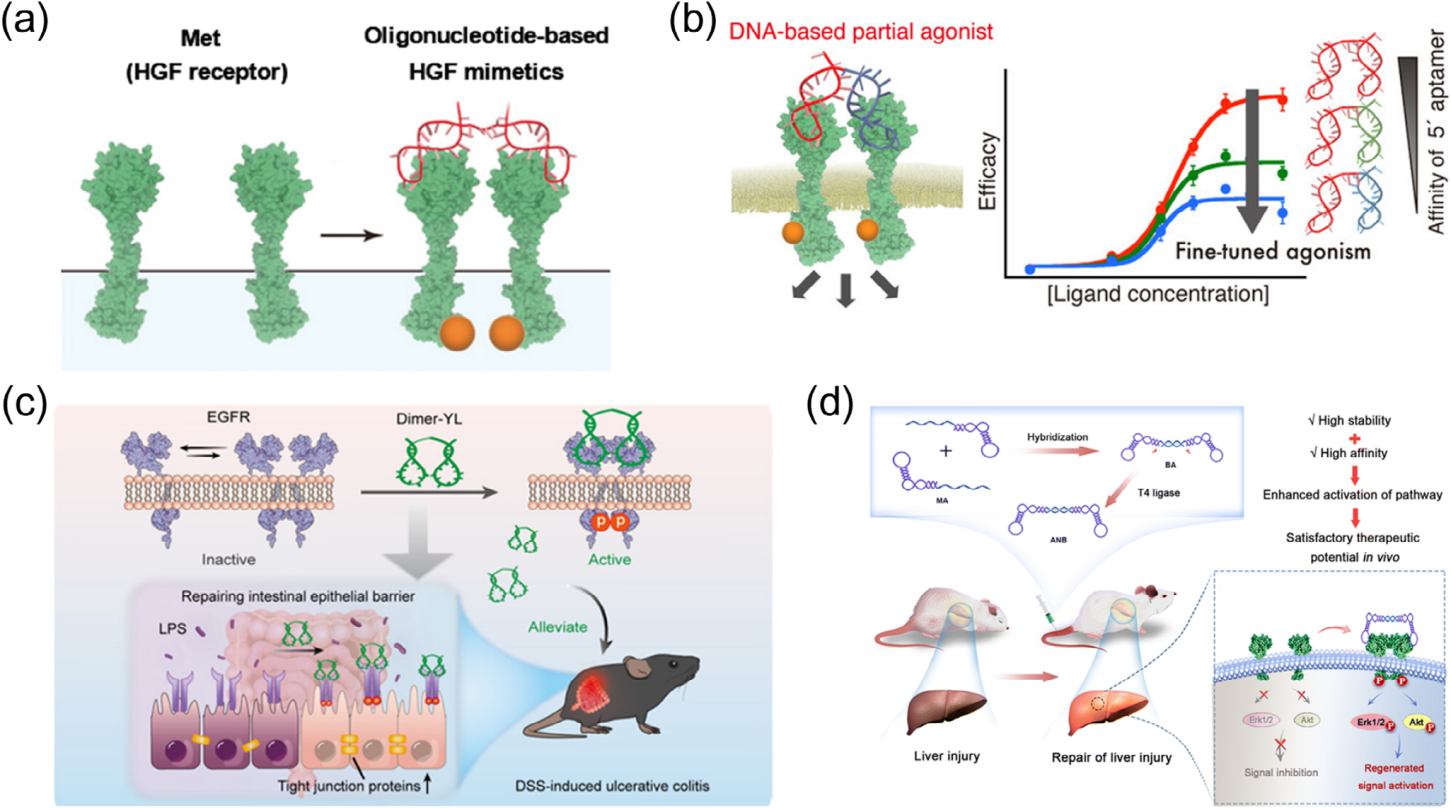
Engineering of DNA‐Based Bivalent Agonists for Targeted Receptor Activation in in vivo Therapeutic Applications. a) A DNA‐based bivalent agonist mimics HGF to induce c‐Met receptor dimerization and activation, regulating downstream signaling. Reproduced with permission.^[^ ^80^ ^]^ Copyright 2016, John Wiley and Sons. b) A fine‐tuned partial agonist is constructed by tethering 5′‐terminal aptamer monomers with varying affinities, enabling controlled receptor activation with adjustable signaling intensity. Reproduced with permission.^[^ ^82^ ^]^ Copyright 2021, John Wiley and Sons. c) A bivalent aptamer‐based DNA agonist targets EGFR, enhancing receptor phosphorylation and promoting intestinal epithelial barrier repair, effectively alleviating dextran sulfate sodium salt (DSS)‐induced UC in vivo. Reproduced with permission.^[^ ^85^ ^]^ Copyright 2024, American Chemical Society. d) A circularized bivalent aptamer enhances receptor stability and activation of regenerative signaling pathways, facilitating liver injury repair in vivo. Reproduced with permission.^[^ ^86^ ^]^ Copyright 2023, Royal Society of Chemistry.

RTKs and cytokine receptors rely on ligand‐induced dimerization as a fundamental activation mechanism, mediating signal transduction across the membrane. Inspired by this natural dimeric process, bivalent aptamer‐based agonists have been constructed from nucleic acid scaffolds to conjugate two receptor‐binding aptamers.^[^ ^76^, ^77^ ^]^ To achieve effective receptor engagement, high‐affinity aptamers targeting receptor extracellular domains are selected and integrated into a bivalent DNA scaffold (**Figure** **4**). This scaffold is designed to fold into a dimeric conformation, ensuring precisely positioned aptamers to facilitate receptor dimerization and downstream signaling activation. Compared to conventional protein growth factors, DNA‐based agonists exhibit superior resistance to enzymatic degradation, high receptor specificity, and the ability to fine‐tune receptor dimerization through sequence modifications.^[^ ^78^ ^]^

Building on this principle, several studies have validated the efficacy of DNA bivalent agonists in receptor activation. Allen et al. designed a bivalent DNA aptamer that effectively dimerized vascular endothelial growth factor receptor‐2 (VEGFR2), successfully promoting angiogenesis through receptor dimerization and activation.^[^ ^79^ ^]^ Similarly, Sando et al. engineered dimeric DNA aptamers mimicking hepatocyte growth factor (HGF), which activated Met receptors, regulating proliferation and migration with tunable receptor engagement (Figure 4a).^[^ ^80^ ^]^ A comparable bivalent aptamer targeting fibroblast growth factor receptor (FGFR1) effectively maintained the pluripotency and self‐renewal of human induced pluripotent stem cells (hiPSCs).^[^ ^81^ ^]^ One of the key advantages of DNA‐based agonists is their intrinsic tunability. Because aptamers are entirely DNA‐based, their interaction affinity with receptors can be precisely modulated by altering their sequence composition. This enables the rational design of partial agonists, which selectively modulate receptor activity rather than fully activating signaling pathways. Sando et al. further leveraged this feature by deleting the stem sequence at the 5'‐end of one of the two aptamers, reducing its affinity and fine‐tuning receptor dimerization propensity, thereby achieving graded activation with minimized off‐target effects (Figure 4b).^[^ ^82^ ^]^

Beyond in vitro validation, DNA bivalent agonists have demonstrated therapeutic efficacy in vivo, highlighting their clinical translation potential. These bivalent agonists designed for growth factor receptor activation have exhibited therapeutic efficacy in mitigating Fas‐induced fulminant hepatitis, where their high nuclease resistance and hepatic localization facilitated treatment.^[^ ^83^ ^]^ Consistent with these findings, Ito et al. utilized HGF aptamer agonists to prevent postoperative peritoneal adhesion (PPA) by enhancing fibrinolysis and inhibiting mesothelial‐mesenchymal transition (MMT).^[^ ^84^ ^]^ Similarly, Li et al. developed Dimer‐YL, a bispecific aptamer agonist targeting epidermal growth factor receptor (EGFR), which exhibited therapeutic effects in ulcerative colitis (UC) models (Figure 4c).^[^ ^85^ ^]^ To further enhance in vivo stability and efficacy, cyclization strategies have been employed. Circularization of aptamers protects against exonuclease degradation, significantly prolonging functional half‐life. Yang et al. developed circular bispecific aptamers (CBAs), which stabilized Met receptor activation, resulting in enhanced repair of acetaminophen (APAP)‐induced liver injury in mice (Figure 4d).^[^ ^86^ ^]^ Our group has further advanced this concept, engineering a circular DNA agonist to activate MET signaling, promoting angiogenesis and diabetic ulcer regeneration.^[^ ^87^ ^]^

In conclusion, DNA bivalent agonists represent a promising class of artificial ligands for highly selective receptor activation. They offer significant advantages over traditional protein‐based therapeutics, including superior stability, tunable activity, and reduced potential for systemic toxicity. These properties make DNA‐based agonists attractive candidates for regenerative medicine and targeted therapies.

### 4.2 DNA‐Scaffolded Polyvalent Ligands for Receptor Assembly

**Figure 5:**
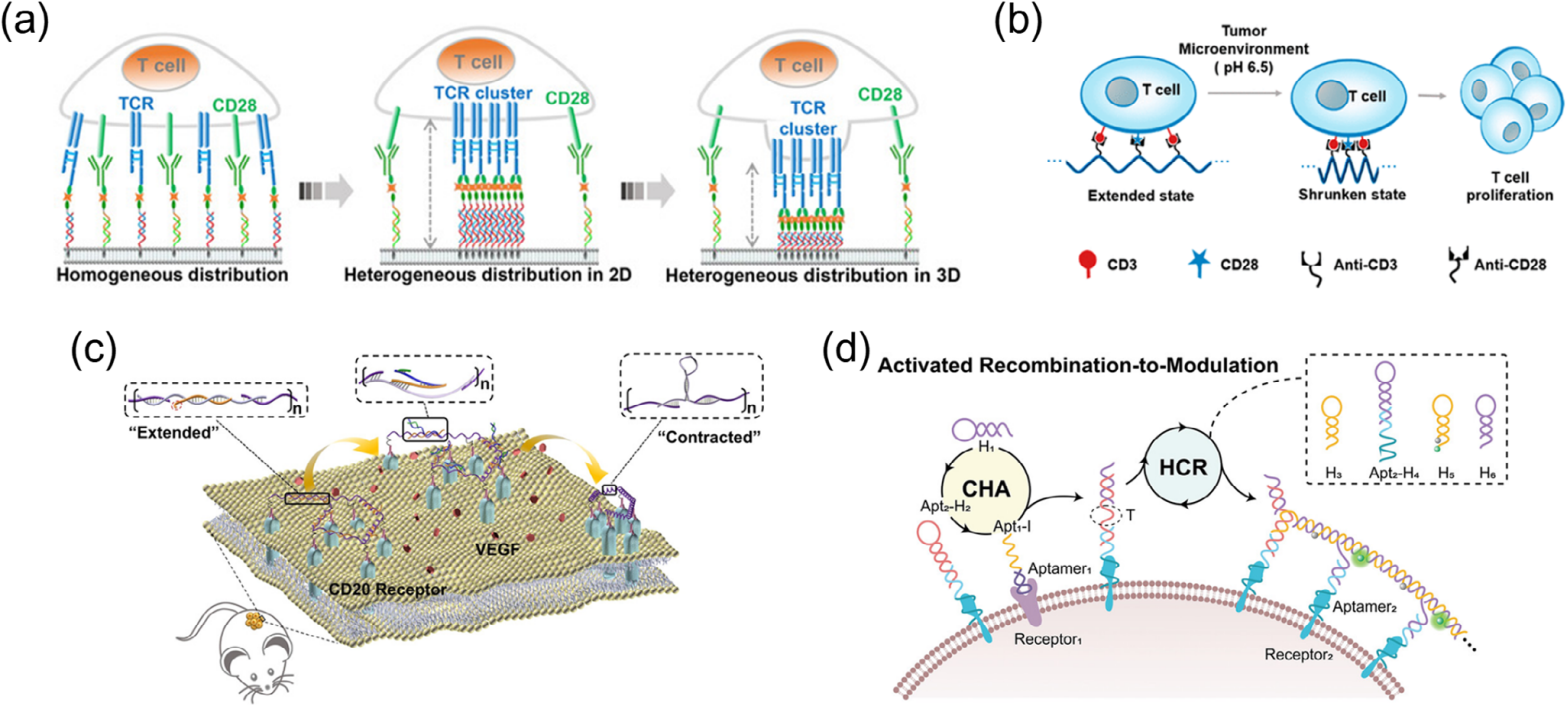
Polyvalent Receptor Assembly for Signal Modulation via DNA Scaffolding. a) DNA‐scaffolded artificial APCs on red blood cells (RBCs) control the lateral and vertical organization of pMHC and CD28 ligands to optimize T‐cell activation. Reproduced with permission.^[^ ^91^ ^]^ Copyright 2020, John Wiley and Sons. b) pH‐responsive iDNS modified with anti‐CD3 and anti‐CD28 undergoes reversible contraction in the tumor microenvironment, dynamically modulating TCR clustering and enhancing T‐cell proliferation for improved immunotherapy. Reproduced with permission.^[^ ^92^ ^]^ Copyright 2022, American Chemical Society. c) VEGF‐driven SMARC utilizes stretchable DNS to selectively cluster CD20 receptors on tumor cells, inducing apoptosis while sparing normal B cells. Reproduced with permission.^[^ ^93^ ^]^ Copyright 2023, American Chemical Society. d) ARM system integrates CHA and HCR to amplify receptor‐mediated signaling for selective imaging and targeted modulation of membrane receptor activity. Reproduced with permission.^[^ ^96^ ^]^ Copyright 2023, John Wiley and Sons.

While ligand‐induced dimerization is a well‐established mechanism for receptor activation, orchestrating higher‐order receptor assembly offers a sophisticated regulatory layer to amplify or fine‐tune signal strength.^[^ ^88^ ^]^ However, natural protein ligands are often limited to monovalent or dimeric forms, restricting their ability to control receptor density and signal amplification. DNA scaffolds emerge as a powerful and programmable alternative. Leveraging DNA's inherent structural programmability, researchers can design multivalent DNA ligands that precisely arrange ligands in space, enabling systematic control over receptor oligomerization and subsequent signaling intensity. This approach expands the toolkit for synthetic receptor modulation, offering unprecedented control over receptor interactions (**Figure** **5**).

To demonstrate this principle, Zhao et al. utilized rolling circle amplification (RCA) to generate DNA scaffolds that organized anti‐CD20 antibodies into multivalent assemblies. This strategy effectively enhanced CD20 clustering on B cells, leading to a significant improvement in apoptotic activity compared to monovalent antibodies. This work underscored the critical role of ligand valency in receptor signaling.^[^ ^89^ ^]^ Similarly, Vale et al. employed DNA‐based synthetic ligands mimicking TCR‐peptide‐major histocompatibility complex (pMHC) interactions. Their findings revealed that T cells exhibit remarkable sensitivity, distinguishing ligands differing by a single base pair, highlighting the influence of ligand affinity on receptor clustering and phosphorylation.^[^ ^90^ ^]^ Further expanding this concept, Liu et al. engineered DNA‐scaffolded artificial antigen‐presenting cells (APCs). By controlling ligand spacing to cluster pMHC and CD28 antibodies (aCD28) at the nanoscale, they optimized T‐cell activation and achieved enhanced in vivo antitumor responses (Figure 5a).^[^ ^91^ ^]^

Beyond static ligand organization, dynamic DNA scaffolds introduce stimulus‐responsive receptor clustering. Shi et al. developed a pH‐sensitive interlocked DNA nano‐spring (iDNS) that reconfigures in the acidic tumor microenvironment. This reconfiguration modulates TCR clustering, enhancing CD8^+^ T cell activation at optimal ligand spacing (Figure 5b).^[^ ^92^ ^]^ In a similar strategy, Liu et al. developed a VEGF‐driven Selective Manipulation of Receptor Clustering (SMARC), employing a DNA nano‐spring (DNS) contraction system to selectively organize the receptors on tumor cells. This targeted approach effectively induced apoptosis in malignant cells while minimizing off‐target effects on healthy tissues (Figure 5c).^[^ ^93^ ^]^ More recently, they developed a DNA‐based “safety catch” system utilizing RCA‐generated contractile nanostring. This system induces PD‐L1 clustering and subsequent lysosomal degradation, improving immunotherapy specificity and reducing adverse immune effects.^[^ ^94^ ^]^

Programmable DNA scaffolds also enable selective receptor modulation through amplification‐based strategies. HCR and CHA, as common signal amplification strategies in DNA nanotechnology, can achieve the directed arrangement and clustering regulation of membrane receptors through in situ dynamic self‐assembly on the cell membrane surface under a defined initiator.^[^ ^16^, ^95^, ^96^ ^]^ Wang et al. employed HCR to drive receptor clustering, demonstrating tunable control over cell migration and proliferation (Figure 5d).^[^ ^95^ ^]^ To enhance specificity, Liu et al. developed an activated recombination‐to‐modulation (ARM) cascade strategy. This system leverages CHA and HCR in a cooperative manner to induce receptor reorganization, effectively blocking Met signaling and inhibiting tumor migration.^[^ ^96^ ^]^

Collectively, these studies highlight the versatility of multivalent DNA scaffolds in promoting receptor clustering and activation to control over cellular signaling for targeted therapeutic interventions.

### 4.3 DNA Origami for Spatial Organization of Receptors

Compared to polyvalent DNA scaffolds that cluster receptors by tethering ligands in predefined multivalent formats, DNA origami introduces an additional level of precision through programmable spatial control at the nanoscale. Beyond merely enhancing valency, origami structures provide a rigid, addressable framework to fine‐tune ligand positioning, spacing, and orientation, enabling systematic exploration of spatial determinants in receptor activation and downstream signaling.

**Figure 6:**
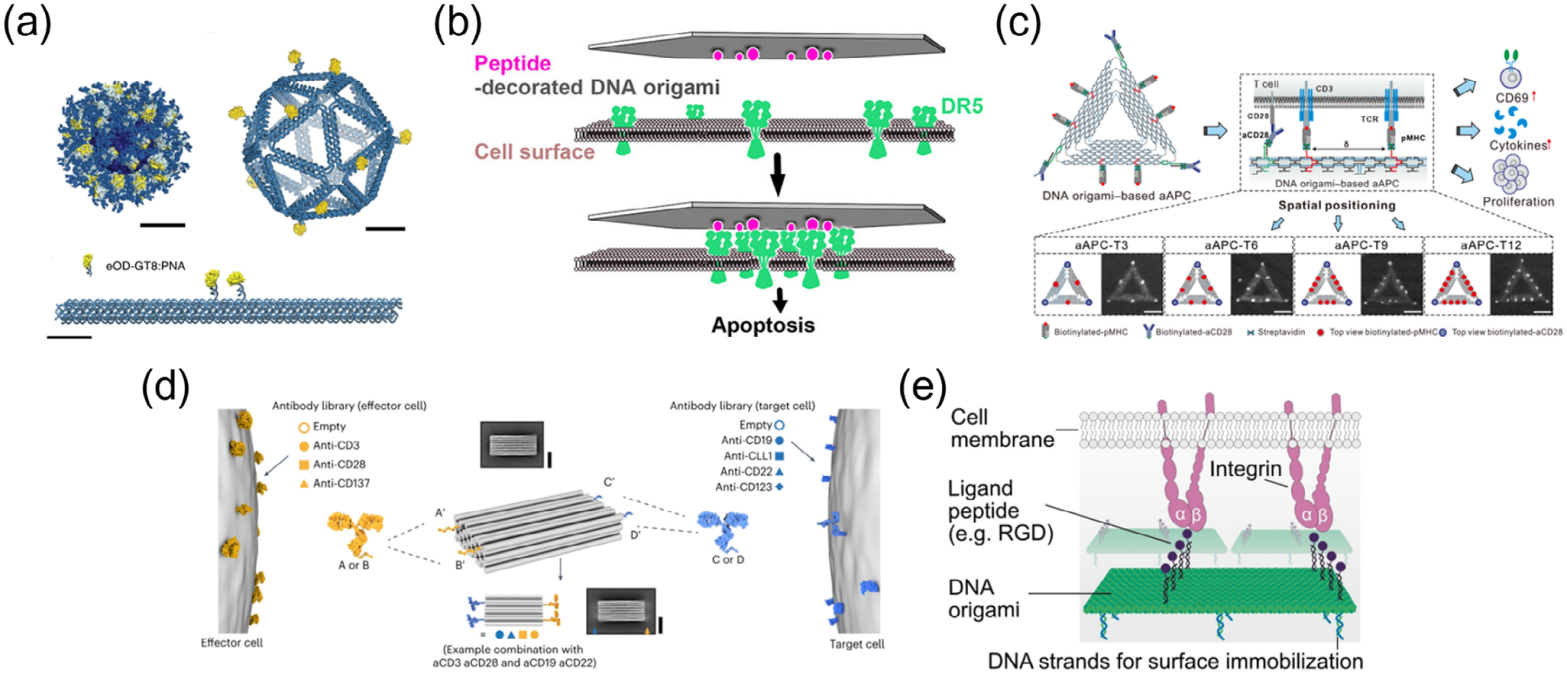
DNA Origami Scaffolds for Programmable Spatial Organization of Protein and Peptide Ligands. a) DNA origami‐based scaffold organizes the copy number, spatial distribution, and presentation dimensionality of HIV immunogens (eOD‐GT8) to optimize B‐cell activation. Reproduced with permission.^[^ ^98^ ^]^ Copyright 2020, Springer Nature. b) DNA origami templates spatially arrange TRAIL‐mimicking peptides to induce death receptor 5 (DR5) clustering, triggering apoptotic signaling in tumor cells. Reproduced with permission.^[^ ^99^ ^]^ Copyright 2021, American Chemical Society/CC‐BY 4.0. c) A DNA origami‐based artificial APC utilizes nanoscale spatial positioning of pMHC and co‐stimulatory ligands (aCD28) to enhance T cell activation and proliferation. Reproduced with permission.^[^ ^105^ ^]^ Copyright 2022, American Association for the Advancement of Science/CC‐BY 4.0. d) A programmable DNA origami‐based T cell engager assembles IgG antibodies, F(ab) fragments, and scFv fragments onto a DNA scaffold, enabling modular control of T cell‐mediated cytotoxicity. Reproduced with permission.^[^ ^106^ ^]^ Copyright 2023, Springer Nature. e) A DNA origami‐based microfluidic platform presents spatially controlled adhesion peptides (e.g., RGD) to study circulating tumor cell (CTC) interactions with endothelial cells and the extravasation process. Reproduced with permission.^[^ ^110^ ^]^ Copyright 2024, John Wiley and Sons.

Over the past decade, DNA origami has been widely utilized to study receptor clustering effects by assembling natural ligands with defined nanoscale spacing.^[^ ^46^, ^47^ ^]^ Teixeira et al. developed a DNA origami‐based nano‐caliper to accurately position ephrin‐A5 ligands for EphA2 receptor clustering, revealing that optimal ligand spacing significantly enhanced EphA2 phosphorylation and suppressed breast cancer cell invasiveness.^[^ ^97^ ^]^ Similarly, Bathe et al. employed DNA origami to pattern the clinically relevant HIV immunogen eOD‐GT8, demonstrating that precise ligand number and spacing maximized B cell activation, with antigen spacing of 25–30 nm proving most effective (**Figure** **6**a).^[^ ^98^ ^]^ Högberg et al. further extended this concept to tumor necrosis factor‐related apoptosis‐inducing ligand (TRAIL), showing that a 5 nm spacing between TRAIL ligands optimized receptor clustering and apoptotic signaling in tumor cells (Figure 6b).^[^ ^99^ ^]^ Beyond these examples, DNA origami has been employed to organize a wide range of receptor‐ligand interactions, including PD‐1 receptor ligands,^[^ ^100^ ^]^ death receptor ligands (FasL, TRAIL, and CD95L),^[^ ^101^ ^]^ insulin receptor ligands,^[^ ^102^ ^]^ and Notch receptor ligands,^[^ ^103^ ^]^ enabling detailed investigations into receptor‐mediated signaling and its biomedical applications.

Moreover, DNA origami has also been applied to highly controlled T cell activation by spatially arranging TCR ligands. Sevcsik et al. demonstrated that a minimum 20 nm pMHC spacing on DNA origami was required for TCR signaling.^[^ ^104^ ^]^ Pei et al. further optimized artificial APCs using DNA origami scaffolds decorated with pMHC and co‐stimulatory ligands, revealing that high‐density ligand presentation enhanced T cell activation and tumor suppression (Figure 6c).^[^ ^105^ ^]^ Expanding this concept, Dietz et al. designed a DNA origami‐based programmable T cell engager that modularly assembled IgG antibodies, F(ab) fragments, and scFvs, improving T cell‐mediated tumor killing (Figure 6d).^[^ ^106^ ^]^ Liu and Sun et al. further demonstrated that nanoscale TCR ligand arrangement and local enrichment enhanced T cell activation, identifying an optimal spacing of ∼38 nm between TCR and co‐stimulatory ligands for robust T cell expansion and antitumor responses.^[^ ^107^ ^]^

Beyond immune modulation, DNA origami has been integrated into biomimetic nanoarrays to study receptor‐ligand interactions in cancer and cell adhesion. Palma et al. fabricated integrin‐EGF nanoarrays to dissect nanoscale ligand crosstalk, revealing ligand density‐dependent effects on melanoma cell proliferation.^[^ ^108^ ^]^ Later, this strategy was applied to study how EGF ligand spacing regulates EGFR activation.^[^ ^109^ ^]^ Recently, Niemeyer's team developed a microfluidic DNA origami‐based system to mimic the cancer cell extravasation process (Figure 6e).^[^ ^110^ ^]^ They found that specific adhesion peptides, such as RGD (arginine‐glycine‐aspartic acid) and IDS (isoleucine‐aspartic acid‐serine), enhanced breast cancer cell extravasation efficiency when presented at a 30 nm spacing, providing insights into cancer metastasis.

**Figure 7:**
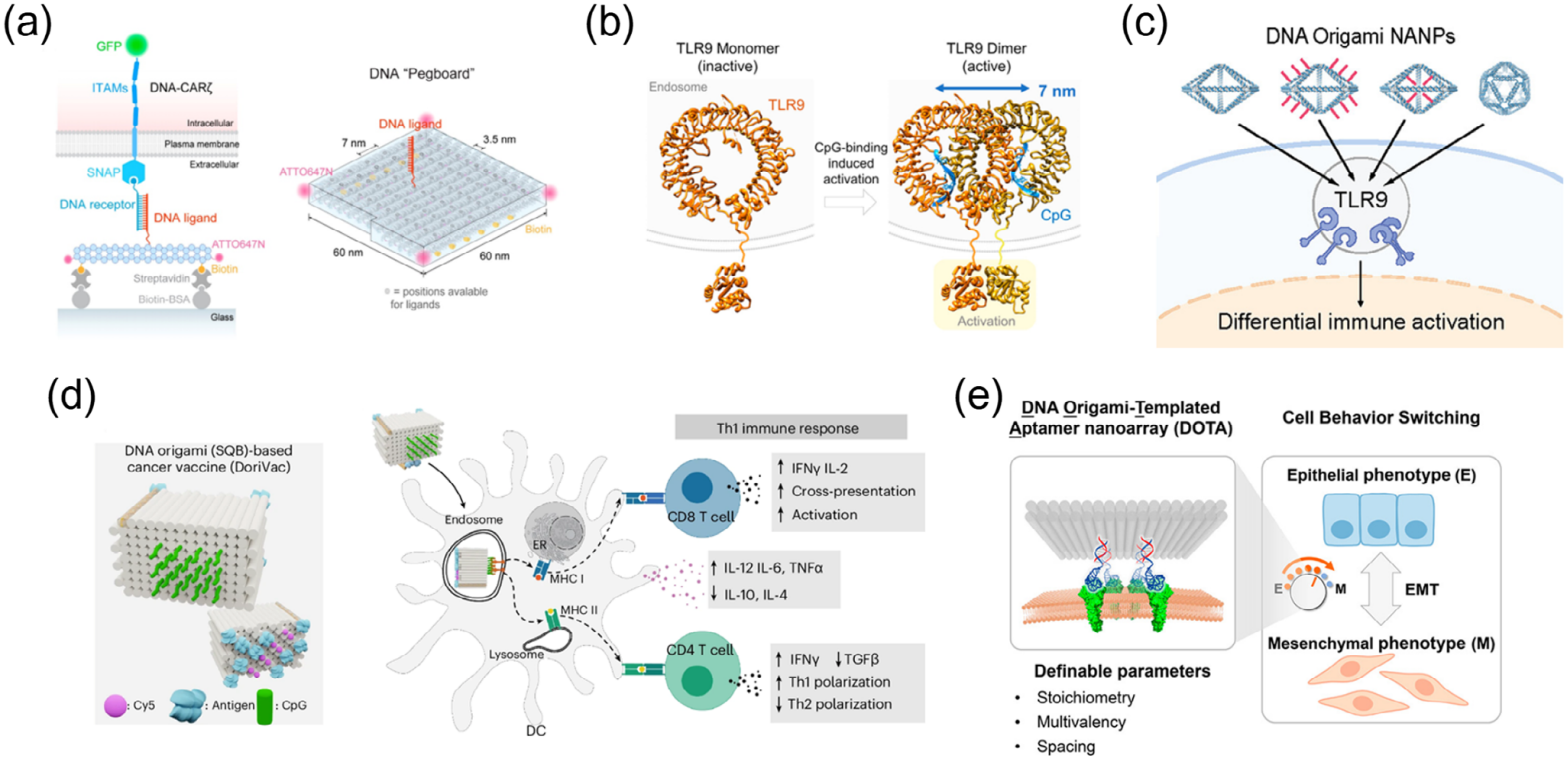
DNA Origami for Spatially Programmed Nucleic Acid Ligands to Modulate Receptor Clustering and Signaling. a) DNA origami‐based molecular pegboards enable nanoscale ligand patterning to control T‐cell activation, modulating receptor clustering, signal sensitivity, and activation kinetics. Reproduced with permission.^[^ ^111^ ^]^ Copyright 2021, National Academy of Science. b) DNA origami nanoscaffolds display CpG motifs with defined spatial arrangements to optimize TLR9 activation. Reproduced with permission.^[^ ^113^ ^]^ Copyright 2022, American Chemical Society. c) Wireframe DNA origami nanoparticles (NANPs) with tunable geometries and CpG copy numbers for programmable control of TLR9‐mediated immune activation. Reproduced with permission.^[^ ^114^ ^]^ Copyright 2022, American Chemical Society d) SQB DNA origami scaffolds presenting CpG oligonucleotide arrays at defined nanoscale spacing to enhance immune responses in cancer vaccines. Reproduced with permission.^[^ ^115^ ^]^ Copyright 2024, Springer Nature. e) DOTA nanoarray for precise spatial control of RTK oligomerization, regulating receptor stoichiometry and signaling dynamics to drive cell fate transitions. Reproduced with permission.^[^ ^116^ ^]^ Copyright 2022, American Chemical Society.

Building upon DNA‐protein conjugates and complementary DNA hybridization, DNA origami offers an advanced approach to accurately program receptor‐ligand interactions. Early strategies relied on SNAP‐tag protein‐DNA conjugation, where DNA hybridization enabled tunable receptor clustering and signal activation. Vale et al. leveraged this concept by designing a DNA origami “pegboard”, which systematically controlled ligand number and spacing to investigate MAP kinase (MAPK) signaling in T cells (**Figure** **7**a).^[^ ^111^ ^]^ The results showed that the spatial arrangement of ligands not only determined the threshold of signal activation but also encoded the time course of signaling activity. In particular, low‐affinity ligands were able to enhance the sensitivity of T cells to signaling when in the vicinity of high‐affinity ligands. This approach was further extended to study Fcγ receptor clustering in macrophage phagocytosis, providing key insights into immune receptor‐mediated effector functions.^[^ ^112^ ^]^

Beyond complementary DNA interactions, immune receptor regulation via cytosine‐phosphate‐guanosine (CpG) DNA spatial patterning has further demonstrated the significance of nanoscale ligand organization. Bastings et al. applied DNA origami to Toll‐like receptor 9 (TLR9), a key immune sensor recognizing pathogen‐derived CpG motifs, revealing that a 7 nm CpG spacing maximized receptor activation, aligning with its active dimer structure (Figure 7b).^[^ ^113^ ^]^ Expanding on this, Bathe et al. developed wireframe DNA origami scaffolds to control CpG ligand number, geometry, and spacing, demonstrating that these parameters modulate TLR9 activation and immune responses (Figure 7c).^[^ ^114^ ^]^ More recently, Shih et al. refined this approach by using a square‐block (SQB) DNA origami template to vary CpG spacing from 2.5 to 7.0 nm, identifying that a 3.5 nm CpG spacing optimized Th1 polarization and T cell activation (Figure 7d).^[^ ^115^ ^]^ These findings highlight the critical role of nanoscale ligand positioning in immune regulation and vaccine development.

While CpG‐based DNA origami primarily targets immune receptors, our group introduced a novel approach, the DNA origami‐templated aptamer nanoarray (DOTA), to regulate receptor spatial clustering and study its impact on cellular signaling and behavior (Figure 7e).^[^ ^116^ ^]^ Unlike DNA hybridization‐based strategies, DOTA employs aptamer‐functionalized origami scaffolds for programmable RTK oligomerization, offering tunable activation with high specificity and structural stability. By mimicking natural receptor clustering while enabling artificial control, this platform regulates key cellular processes such as epithelial‐mesenchymal transition (EMT). As a versatile system, DOTA can be adapted to any receptor through aptamer selection and optimization, providing a universal tool for mechanistic studies and precision control of receptor‐mediated cell responses.

In conclusion, DNA origami has redefined receptor spatial organization by enabling precise control over the presentation of different ligand types (e.g., proteins, peptides, CpG motifs, and aptamers), to fine‐tune receptor clustering and cellular responses. By decoupling variables such as ligand spacing, valency, and geometry, DNA origami bridges molecular‐scale engineering with cellular signaling, offering transformative strategies for cell fate manipulation, cancer immunotherapy, and vaccine development.

### 4.4 Dynamic DNA Nanodevices Customizing Receptor Responsiveness to Molecular Cues

Reprogramming cell surface receptors to respond to molecular cues is a key challenge in synthetic biology and therapeutics. Traditional approaches, such as CARs and CID systems, require genetic modifications, posing risks of biosafety, immune rejection, and limited adaptability.^[^ ^63^, ^117^ ^]^ Recent advances in FNA‐driven dynamic DNA reactions enable programmable receptor responses to proteins, small molecules, ions, gases, and nucleic acids, offering highly controlled cellular signaling and therapeutic interventions without altering endogenous receptors.

#### 4.4.1 Protein‐Mediated Receptor Activation

**Figure 8:**
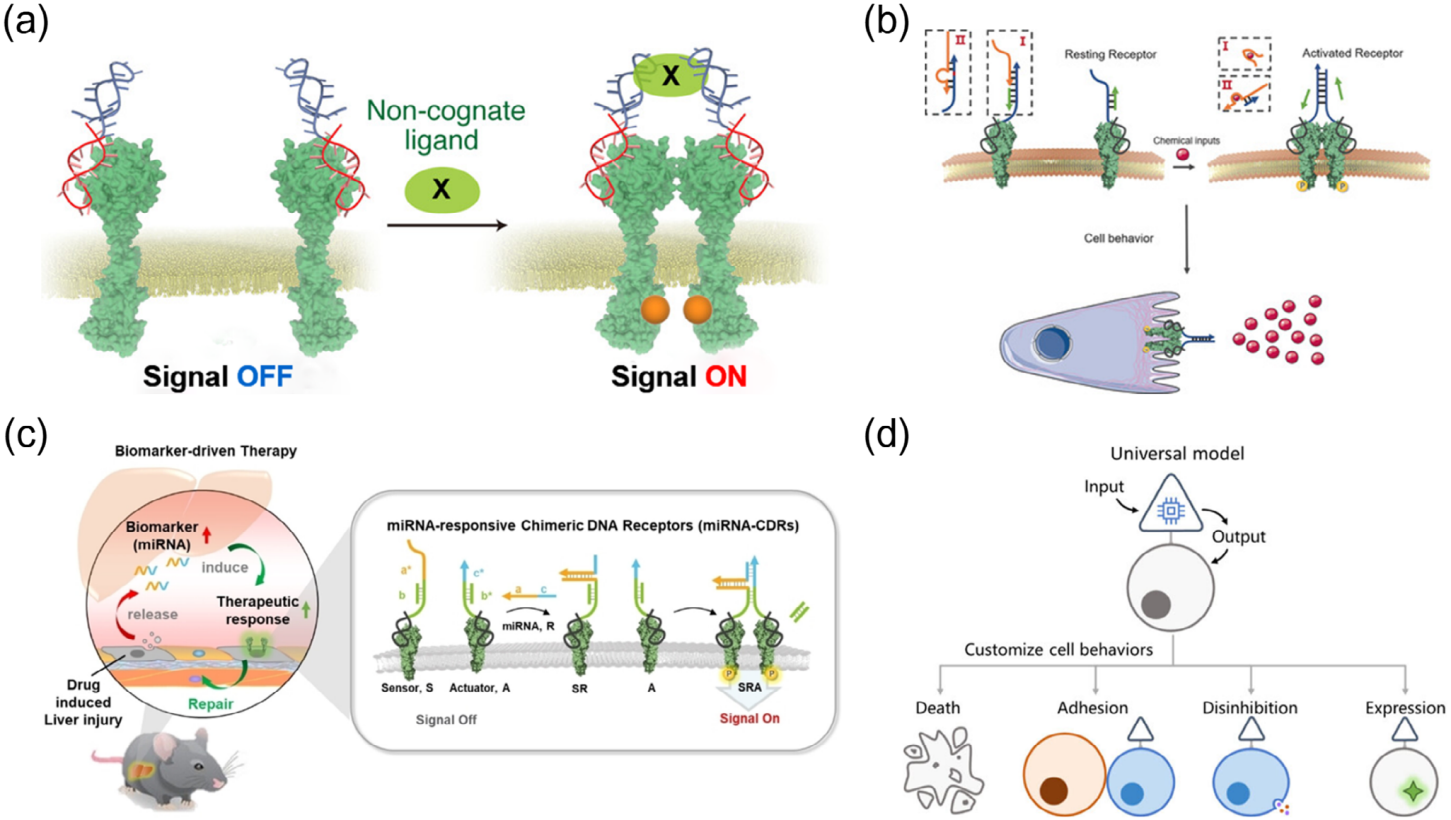
Customizing Receptor Responsiveness to Molecular Cues with Dynamic DNA Nanodevices. a) DRIPaR reprograms c‐Met dimerization by utilizing bispecific aptamers that simultaneously bind two c‐Met receptors and a user‐defined non‐cognate ligand, enabling tunable receptor activation. Reproduced with permission.^[^ ^17^ ^]^ Copyright 2017, American Chemical Society. b) D‐CID is a non‐genetic approach that controls receptor dimerization and modulates cell behaviors in response to chemical inputs such as ATP, histidine, or zinc ions. Reproduced with permission.^[^ ^18^ ^]^ Copyright 2018, John Wiley and Sons. c) miRNA‐CDR is a biomarker‐driven therapeutic nanodevice that detects extracellular miRNAs and triggers receptor dimerization via dynamic DNA strand assembly, activating downstream signaling for tissue repair. Reproduced with permission.^[^ ^119^ ^]^ Copyright 2023, John Wiley and Sons. d) A universal DNA sensing toolbox integrates DNA origami and molecular switching to equip cells with programmable signal sensing and behavioral reprogramming capabilities. Reproduced with permission.^[^ ^121^ ^]^ Copyright 2023, American Chemical Society.

Proteins serve as fundamental activators of receptor signaling, yet rewiring receptors to respond to a new protein typically requires complex genetic modification to introduce recognition domains. Sando et al. proposed a DNA aptamer‐mediated receptor reprogramming strategy, termed DRIPaR, which utilized bispecific DNA aptamers that simultaneously bound RTKs and external protein stimuli to induce receptor dimerization and activation (**Figure** **8**a).^[^ ^17^ ^]^ This modular approach enabled different proteins, such as thrombin and platelet‐derived growth factor (PDGF), to serve as switchable activators of RTKs, regulating cell proliferation and migration without permanent receptor modifications.

#### 4.4.2 Small Molecule or Ion Responsiveness

Small molecules and ions can also serve as crucial environmental cues that can dynamically regulate receptor activity through DNA‐mediated mechanisms. Leveraging target‐recognition aptamers or cofactor‐responsive DNAzymes to induce dynamic DNA assembly combines aptameric sensing or DNAzyme‐based cleavage with the strand displacement reaction, our group developed a DNA‐mediated chemically induced dimerization (D‐CID) system, where ATP binding to its cognate aptamer triggered strand displacement, leading to complementary DNA‐driven receptor dimerization (Figure 8b).^[^ ^18^ ^]^ In contrast, Zn^2^⁺ and histidine activated the cognate respective DNAzymes, cleaving DNA strands to induce receptor activation. This approach enables orthogonal receptor regulation, allowing multiple signals to independently govern cellular behaviors.

#### 4.4.3 Gasotransmitter Responsiveness

Gasotransmitters, such as hydrogen sulfide (H₂S), present unique challenges in receptor engineering due to their high diffusivity and transient bioactivity. To address this, our group recently developed Gas‐responsive artificial DNAzyme‐based switches (GRAS), which employ H₂S‐mediated decaging of DNAzymes to activate receptor dimerization.^[^ ^118^ ^]^ Stimuli‐responsive moieties (specifically, H₂S‐responsive azide groups) are incorporated into the phosphorothioate backbone of the DNAzyme at cofactor binding locations within the catalytic core region. H₂S reduces these azide groups, uncaging the DNAzyme and initiating substrate cleavage, which ultimately leads to receptor activation. By linking gas sensing with receptor activation, this platform provides targeted control over cell migration and proliferation, demonstrating potential applications in gas‐responsive cell therapies.

#### 4.4.4 Nucleic Acid Biomarker‐Driven Receptor Activation

Nucleic acid biomarkers, particularly microRNAs (miRNAs), are critical indicators of disease and hold significant potential for controlling therapeutic interventions. However, most current receptor engineering strategies lack intrinsic miRNA recognition mechanisms. To address this gap, our group developed a miRNA‐responsive chimeric DNA receptor (miRNA‐CDR), integrating aptamer‐anchored DNA nanodevices that sense extracellular miRNAs and induce receptor activation through a miRNA‐driven strand displacement reaction (miRNA‐CSD) (Figure 8c).^[^ ^119^ ^]^ Upon miRNA binding, miRNA‐CSD triggers receptor dimerization and intracellular signaling activation, enhancing the repair of drug‐induced acute liver injury. This biomarker‐driven receptor activation provides a versatile strategy for precision medicine, enabling in situ tissue regeneration and targeted therapeutic interventions.

#### 4.4.5 Multi‐Input Responsiveness

Beyond responding to single inputs, DNA nanodevices can be designed to intelligently respond to multiple environmental cues. Wang et al. constructed DNA nanodevices responsive to environmental inputs, such as pH and ATP, using pH‐responsive i‐motifs and ATP‐binding aptamers to selectively assemble Met and CD71 receptors, forming AND logic gates for tumor microenvironment regulation.^[^ ^120^ ^]^ Recently, Yin et al. introduced an integrated DNA‐based sensing toolkit, utilizing a triangular prism‐shaped DNA origami scaffold to enable signal sense‐trigger‐respond systems for cell programming (Figure 8d).^[^ ^121^ ^]^ By incorporating signal converters and functional molecules, this toolkit provides a programmable platform for cellular response modulation.

In conclusion, dynamic DNA nanodevices offer a versatile approach to rewire receptor signaling to respond to a wide range of molecular cues, from proteins and small molecules to gaseous cues and miRNAs, with modularity and tunability. This strategy presents a promising avenue for developing smart therapeutics and biomarker‐driven targeted medicine.

### 4.5 Light‐Responsive DNA Nanodevices for Spatiotemporal Receptor Regulation

Engineering receptor signaling with light offers an unparalleled approach to dissect cellular processes and develop precision therapies. Optical control provides non‐invasive, high‐resolution, and spatiotemporal regulation of receptor function, enabling dynamic modulation of cell behavior in real time. By integrating photocleavable linkers, photo‐crosslinking agents, and optically tunable nanomaterials, researchers have developed versatile DNA‐based systems to achieve light‐triggered receptor activation, advancing both mechanistic studies and therapeutic applications.

**Figure 9:**
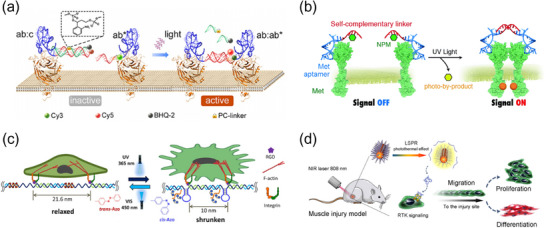
Light‐Responsive DNA Nanodevices for Spatiotemporal Control of Receptor Activation and Cellular Signaling. a) Photo‐controlled DNA assembly enables receptor dimerization and signaling activation on the cell membrane. Reproduced with permission.^[^ ^19^ ^]^ Copyright 2018, John Wiley and Sons. b) Photocaged DNA aptamer with 6‐NPM modification allows non‐genetic regulation of receptor dimerization and signaling activation upon UV exposure. Reproduced with permission.^[^ ^122^ ^]^ Copyright 2021, Royal Society of Chemistry. c) Azobenzene‐modified DNA polymer undergoes reversible conformational changes under UV and visible light, dynamically regulating cell morphology.^[^ ^125^ ^]^ Copyright 2021, John Wiley and Sons. d) NIR‐DA nanodevice releases active DNA agonists via NIR laser activation, inducing c‐Met dimerization to control cell proliferation, differentiation, and muscle regeneration in vivo. Reproduced with permission.^[^ ^20^ ^]^ Copyright 2019, American Chemical Society.

By integrating light‐sensitive elements (e.g., photocleavable groups or azobenzene units) into the DNA structure, it is possible to make it responsive to UV light stimulation. This light‐responsive mechanism enables precise control of conformational changes, assembly and de‐assembly, and functional activation of DNA nanodevices, thereby modulating receptor activation or cellular behaviors that interact with DNA nanodevices.^[^ ^7^ ^]^ Light‐controlled receptor activation has mainly relied on UV‐responsive DNA architectures. Yang et al. designed a photoactivatable DNA assembly where one Met receptor‐recognizing aptamer was blocked by a strand containing a UV‐cleavable o‐nitrobenzyl (PC) linker (**Figure** **9**a).^[^ ^19^ ^]^ Upon UV exposure, the blocking strand was cleaved, initiating strand displacement‐driven receptor dimerization and activation of downstream signaling. Similarly, Sando et al. employed a caged Met aptamer modified with a 6‐nitropiperonyl‐α‐methyl (NPM) group, which prevented hybridization of two complementary strands (Figure 9b).^[^ ^122^ ^]^ UV irradiation removed the caging group, restoring aptamers' terminal strands hybridization function and enabling receptor dimerization. These systems demonstrated photochemical control of receptor activation, allowing dynamic tuning of signaling by adjusting the light input pattern. Expanding on this, Han et al. introduced a photo‐crosslinking DNA system, where a 3‐cyanovinylcarbazole nucleoside enabled covalent crosslinking under UV light, increasing receptor labeling efficiency and enhancing Met receptor inhibition.^[^ ^123^ ^]^

Beyond direct receptor activation, light‐responsive DNA nanodevices have been utilized to regulate cellular morphology and mechanical signaling. Li et al. developed a DNA nanospring‐based ligand that undergoes strand displacement‐driven stretching and contraction, modulating receptor function and controlling PI3K/Rac1‐mediated cellular protrusions.^[^ ^124^ ^]^ Inspired by this, Endo et al. integrated azobenzene‐modified DNA polymers with RGD ligands, constructing a photo‐switchable system that transitions between relaxed and shrunken states in response to UV and visible light (Figure 9c).^[^ ^125^ ^]^ This system induced actin reorganization and cell shape modulation, providing a framework for light‐controlled cellular behavior. Meanwhile, Sando et al. recently developed a light‐responsive DNA aptamer agonist, Met‐azo‐aptamer, designed to reversibly control the activity of the Met receptor. By incorporating azobenzene into the DNA aptamer, they enabled light‐induced switching between active and inactive states of the aptamer, allowing precise temporal control over Met activation and downstream ERK signaling in cells. This tool offers a high‐resolution method to investigate the relationship between RTK activation patterns and cellular functions or fate without requiring genetic modifications.^[^ ^126^ ^]^

However, the above UV‐responsive DNA nanodevices face challenges, including phototoxicity and poor tissue penetration, which restrict their in vivo applications. To address these issues, our group developed the first near‐infrared light‐activated DNA agonist (NIR‐DA) for non‐genetic receptor regulation in deep tissues (Figure 9d).^[^ ^20^ ^]^ This system utilizes gold nanorods (AuNRs) with localized surface plasmon resonance (LSPR)‐mediated photothermal release of pre‐inactivated DNA agonists. Upon NIR exposure, DNA agonists are released, triggering aptamer‐mediated receptor dimerization, cytoskeletal remodeling, and cell polarization‐driven migration. In a murine skeletal muscle injury model, NIR‐induced receptor activation stimulated satellite cell migration, proliferation, and differentiation, accelerating skeletal muscle regeneration in deep tissue.

Together, light‐responsive DNA nanodevices provide an unprecedented level of spatiotemporal precision in receptor control. By integrating programmable DNA architectures with optical modulation, these systems unlock new possibilities for real‐time signal tuning, in vivo regulation of cellular functions, and advanced phototherapeutic interventions.

### 4.6 DNA‐Based Mechano‐Nanodevices to Regulate Receptor‐mediated Signaling

Cells interpret mechanical cues through specialized mechanosensitive receptors that convert extracellular forces into biochemical signals, regulating morphology, signaling pathways, and physiological functions.^[^ ^127^ ^]^ Key players in this process, known as mechanotransduction, include mechano‐gated ion channels (e.g., Piezo1) and cell adhesion receptors (e.g., integrins, cadherins).^[^ ^128^ ^]^ Beyond these, mechanical forces also modulate immune and developmental signaling through receptors like TCRs and Notch, underscoring the pervasive influence of mechanotransduction on cellular behavior.^[^ ^129^ ^]^ Recent advances in DNA nanotechnology now provide a modular, tunable, and programmable means to control mechanotransduction at the molecular scale, opening up new avenues for research and therapeutic intervention.^[^ ^130^ ^]^

DNA‐based mechano‐nanodevices, with their nanoscale spatial resolution, programmability, and customizable mechanical properties, are particularly advantageous for studying and controlling mechanobiological processes.^[^ ^131^ ^]^ These devices can directly engage native mechanoreceptors, providing insights into receptor mechanics and enabling force‐responsive control over downstream cellular signaling.

#### 4.6.1 Integrin‐Mediated Force Signaling

**Figure 10:**
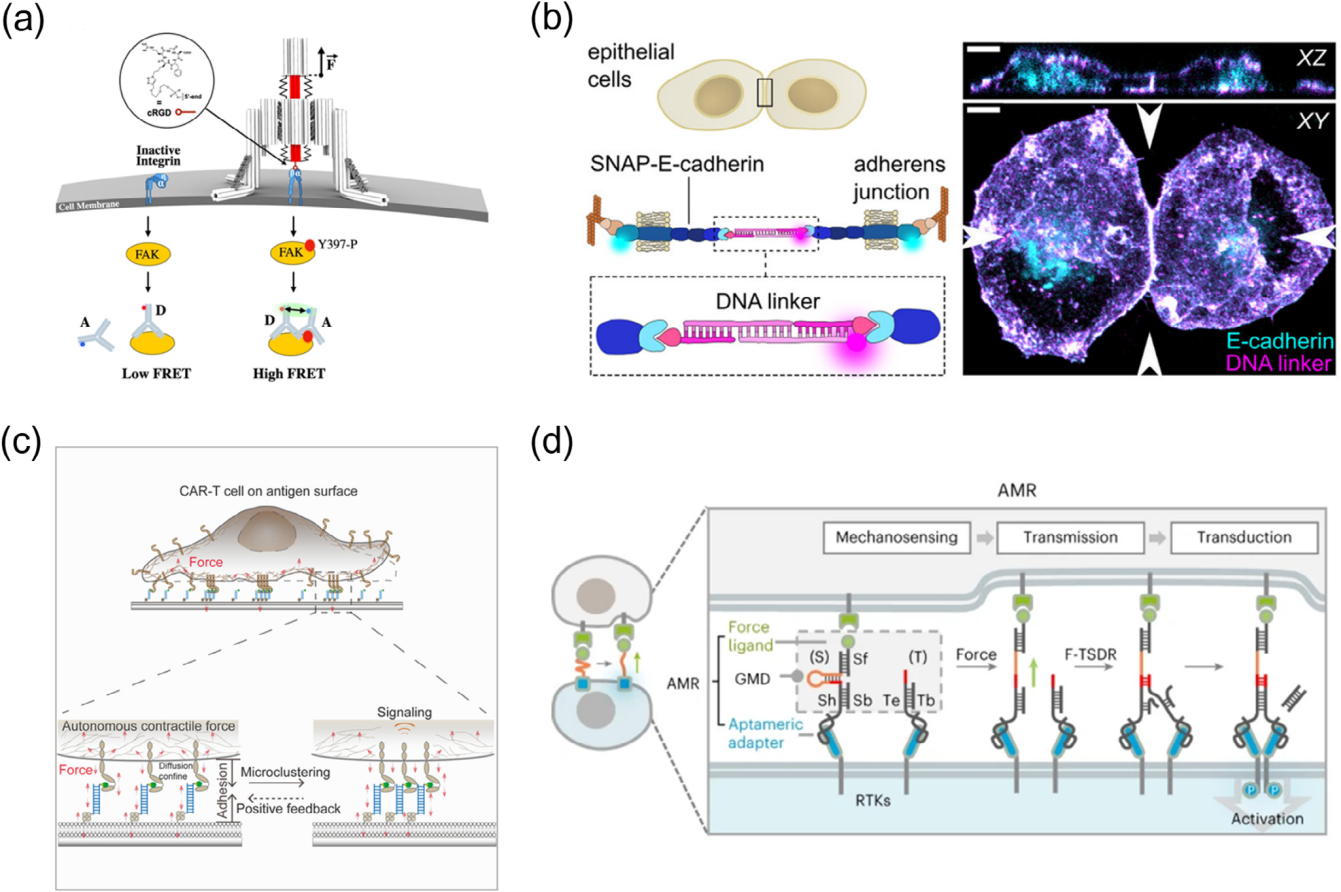
DNA Mechano‐Nanodevices for Modulation of Mechanotransduction. a) A DNA origami Nano‐winch applies tunable pN‐scale forces to activate integrin receptors, enabling controlled mechanotransduction. Reproduced with permission.^[^ ^132^ ^]^ Copyright 2022, Springer Nature/CC BY 4.0. b) A DNA‐E‐cadherin hybrid system modulates cell‐cell adhesion strength through reversible DNA linkers, facilitating controlled epithelial interactions. Reproduced with permission.^[^ ^133^ ^]^ Copyright 2021, American Chemical Society. c) Double‐stranded DNA force sensors regulate CAR receptor activation, demonstrating the mechanical dependence of CAR‐T cell signaling. Reproduced with permission.^[^ ^134^ ^]^ Copyright 2024, Elsevier. d) A DNA‐functionalizd AMR reprograms RTKs to respond to mechanical stimuli via force‐triggered DNA strand displacement. Reproduced with permission.^[^ ^21^ ^]^ Copyright 2024, Springer Nature.

Integrins are critical mechanosensitive receptors mediating cell adhesion and force responses to the extracellular matrix. Bellot et al. developed a DNA origami‐based Nano‐winch capable of autonomously attaching to cell surfaces and exerting piconewton (pN)‐scale mechanical forces on integrin receptors (**Figure** **10**a).^[^ ^132^ ^]^ Functionalized with cRGD peptides, this Nano‐winch selectively targeted integrin receptors on MCF‐7 cells, inducing compressive forces and activating integrin‐FAK signaling. This approach provides a controllable system for investigating receptor mechanics in living cells.

#### 4.6.2 Cell–Cell Adhesive Force Signaling

Mechanical forces also critically regulate cell‐cell interactions, primarily mediated by E‐cadherin proteins. Exploiting DNA dynamic assembly characteristics, Cavalcanti‐Adam et al. engineered DNA‐E‐cadherin hybrids, linking DNA strands to truncated E‐cadherin domains to regulate adhesion strength in epithelial cells (Figure 10b).^[^ ^133^ ^]^ Through DNA strand displacement, cell‐cell adhesion could be dynamically controlled, offering insights into the role of adhesion forces in collective cell behavior.

#### 4.6.3 Force‐Responsive T Cell Activation

In immune cells, mechanical forces modulate T‐cell activation by engaging TCR mechanotransduction pathways, a key factor in optimizing CAR‐T cell efficacy. Xu et al. engineered antigen‐presenting platforms using double‐stranded DNA force sensors, enabling mechanical control over CAR clustering and activation thresholds (Figure 10c).^[^ ^134^ ^]^ By varying DNA duplex length and GC content, they determined a force threshold (≥26 pN) required for efficient CAR activation. These studies underscore the importance of mechanical cues in immune cell engineering and therapeutic efficacy.

#### 4.6.4 De Novo Mechanoreceptor for Cellular Regulation

Existing approaches predominantly rely on genetically engineering or DNA‐based modifying naturally occurring mechanoreceptors, such as synNotch and synCAM (Section 3), limiting adaptability and broad applicability.^[^ ^12^, ^13^ ^]^ To overcome this limitation, recent efforts have focused on constructing fully synthetic mechanotransduction pathways that operate independently of intrinsic receptor mechanosensitivity. In this context, we developed a DNA‐functionalized artificial mechanoreceptor (AMR), a synthetic system designed to confer mechanosensitivity to non‐mechanosensitive receptors, enabling them to respond to mechanical forces (Figure 10d).^[^ ^21^ ^]^ The AMR system integrates a mechanosensing and transmitting DNA (GMD) nanodevice, comprising a sensor module (S) with a stem‐loop structure that unfolds upon force application and a transmitter module (T) that enables DNA strand displacement, triggering receptor dimerization and activation. Due to this modular design, AMRs can transduce mechanical signals derived from cell adhesion molecules (e.g., integrins and E‐cadherin) or membrane‐trafficking proteins (e.g., CI‐M6PR) into targeted activation of selected receptors, including c‐Met and FGFR1. Demonstrating their utility, we successfully applied AMRs to regulate neural stem cell fate via adhesion‐mediated mechanotransduction, illustrating their potential for precision cellular engineering. The AMR approach holds high potential to expand the scope of receptor engineering, providing a versatile platform for mechanotransduction‐based cellular regulation.

In conclusion, the evolution from modulating natural mechanoreceptors to designing programmable, *de novo* mechanosensitive systems represents a significant advance in the field.^[^ ^135^ ^]^ This progress paves the way for adaptive mechanotransduction platforms, enabling customizable and tunable control of cellular behaviors for a wide range of biomedical applications.

### 4.7 Intelligent Modulation of Receptor Signaling by DNA Logic Circuits

Effective receptor modulation is crucial for advancing targeted therapies, yet conventional receptor agonists often suffer from low specificity, off‐target activation, and systemic toxicity due to their widespread presence in both healthy and diseased tissues.^[^ ^136^ ^]^ To overcome these challenges, DNA logic circuits offer a programmable approach for achieving cell‐type‐specific receptor activation. These circuits enable selective recognition and signaling regulation based on molecular logic operations, ensuring that therapeutic effects are focused only on the intended target cells.

DNA logic circuits are designed based on principles of molecular recognition, dynamic strand displacement, and programmable logic operations. These circuits can process a wide range of molecular inputs, including proteins, nucleic acids, and small molecules, showcasing their versatility and programmable potential in receptor control. To design layered dynamic responses for programmable receptor control, these circuits employ DNA recognition modules complementary to specific cell‐surface markers, logic gates that process multiple input signals through Boolean operations, and cascaded reaction networks that translate logic outputs into controlled receptor activation. This capability to integrate diverse molecular inputs ensures precise and context‐dependent cellular modulation, further highlighting the multifunctionality of DNA logic systems in receptor regulation.^[^ ^78b^ ^]^

#### 4.7.1 The SUDA System

**Figure 11:**
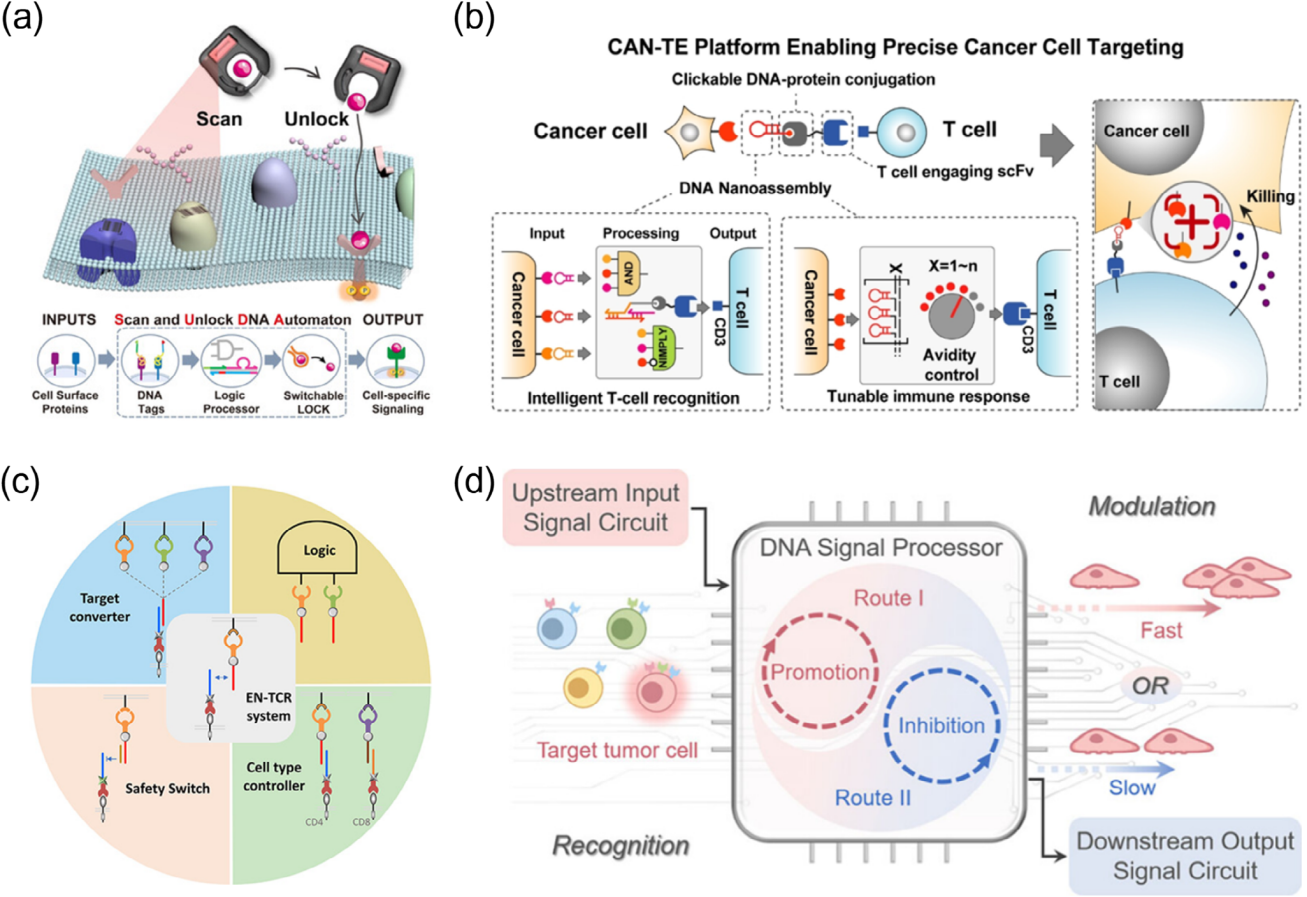
DNA‐based Logic Circuits for Intelligent Receptor Regulation. a) The SUDA platform selectively activates receptor signaling by recognizing specific cell surface protein combinations using DNA logic circuits. Reproduced with permission.^[^ ^54^ ^]^ Copyright 2021, John Wiley and Sons. b) The CAN‐TE system enhances tumor targeting through logic‐based antigen recognition, improving T cell specificity and immune response modulation. Reproduced with permission.^[^ ^53^ ^]^ Copyright 2022, John Wiley and Sons. c) The EN‐TCR modularly assembles antibody‐DNA hybrids for antigen recognition and combinatorial targeting. Reproduced with permission.^[^ ^137^ ^]^ Copyright 2022, American Chemical Society. d) A membrane‐anchored DNA signal processor enables ATP‐controlled tumor recognition and c‐Met receptor modulation via a dual aptamer circuit. Reproduced with permission.^[^ ^138^ ^]^ Copyright 2024, American Chemical Society.

Our group pioneered a DNA‐based engineering strategy called the “scan‐and‐unlock DNA automaton” (SUDA) to achieve highly selective receptor control at the cellular level (**Figure** **11**a).^[^ ^54^ ^]^ The SUDA system incorporates DNA locks that keep ligands in an inactive state, recognition elements that detect multiple surface markers, and a cascaded DNA reaction network that executes conditional activation. By employing different logic operations (e.g., AND, NIMPLY), SUDA allows highly specific discrimination and targeted modulation of any single cell type within a complex mixture of five different cell populations. This selective activation ensures that receptor signaling is triggered only in specific cell types while remaining inert in others, achieving a customizable and adaptable approach to ligand‐receptor modulation.

#### 4.7.2 CAN‐TE Platform

Extending DNA logic circuit‐based strategy to cancer immunotherapy, we developed a chimeric antibody nucleic acid T‐cell engager (CAN‐TE) to enhance the specificity of T‐cell‐mediated tumor targeting (Figure 11b).^[^ ^53^ ^]^ Unlike conventional T‐cell engagers, CAN‐TE employs multi‐biomarker recognition via DNA logic gates, allowing precise control over T‐cell activation in response to multiple tumor‐associated antigens. This system significantly improves T‐cell specificity in recognizing malignant cells while reducing off‐target immune responses, thus enhancing tumor eradication efficiency. The adaptability of CAN‐TE allows it to be tailored for diverse antigen profiles, making it a scalable platform for next‐generation T‐cell‐based cancer immunotherapy.

#### 4.7.3 EN‐TCR Platform

Similarly, Ye et al. developed a nano‐biohybrid DNA engager‐reprogrammed T‐cell receptor (EN‐TCR) to achieve programmable antigen recognition and response tuning (Figure 11c).^[^ ^137^ ^]^ This system consists of signaling (S‐EN) and recognition (R‐EN) modules, which dynamically modulate T‐cell activation through DNA strand hybridization. By implementing AND, OR, and NOT logic gates, EN‐TCR allows combinatorial antigen recognition, ensuring that T cells only respond to tumor‐specific molecular cues.

#### 4.7.4 TCS‐SPP Platform

Beyond T‐cell engineering, programmable logic circuits can be utilized for intracellular signal amplification and pathway control. Bi et al. developed an ATP‐controlled tumor cell‐specific signal processing platform (TCS‐SPP) to regulate cellular behaviors non‐invasively (Figure 11d).^[^ ^138^ ^]^ The system integrates a dual‐aptamer logic circuit for selective tumor recognition, a DNA polymerase‐driven RCA module for signal amplification, and an ATP molecular switch for controlling c‐Met receptor dimerization. This approach enables targeted regulation of tumor cell migration, invasion, and proliferation, demonstrating the potential of DNA logic circuits to fine‐tune oncogenic signaling pathways with high specificity.

In summary, DNA logic circuits provide a programmable strategy for highly selective receptor control, integrating dynamic strand displacement reactions and multi‐layered logic gates to achieve context‐dependent and autonomous cellular regulations. Advances in synthetic biology and DNA computing will drive the development of scalable frameworks for smart cell engineering, paving the way for next‐generation precision therapies.

### 4.8 DNA Nanorobots for Receptor Modulation and Cell Manipulation

**Figure 12:**
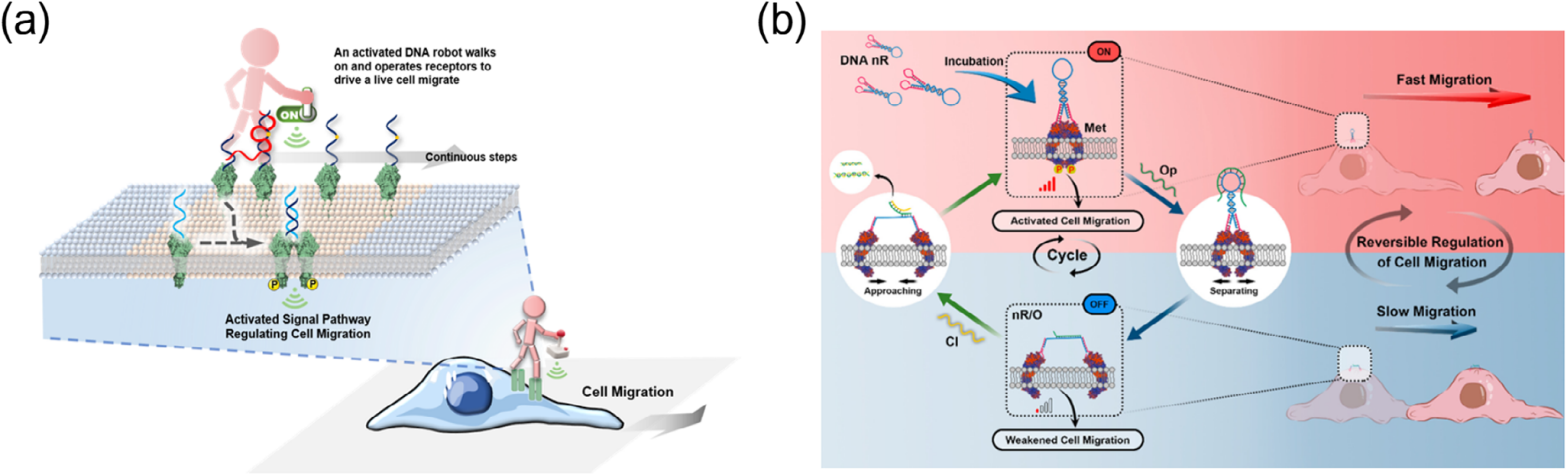
DNA Nanorobots for Receptor Modulation and Cell Manipulation. a) A DNA molecular robot moves autonomously across the cell membrane, using DNAzyme‐driven catalysis to induce receptor dimerization and regulate cell motility. Reproduced with permission.^[^ ^55^ ^]^ Copyright 2021, John Wiley and Sons. b) A reconfigurable DNA nanorobot dynamically switches Met receptor dimerization on and off via DNA strand displacement, enabling reversible control of cell migration. Reproduced with permission.^[^ ^140^ ^]^ Copyright 2023, American Chemical Society.

Building upon the principles of DNA strand displacement cascades, DNA nanorobots offer a highly useful strategy for autonomous receptor regulation, translating molecular interactions into functional cellular responses.^[^ ^139^ ^]^ Conventional DNA walkers are limited to fixed substrates, restricting their applicability in live‐cell environments. To bridge the nanoscale molecular world with large‐scale cellular behavior, our group developed the first example of a membrane‐compatible DNA nanorobot that autonomously navigates the fluidic lipid bilayer and engages surface receptors to modulate cell signaling (**Figure** **12**a).^[^ ^55^ ^]^ Unlike static DNA nanostructures, this dynamic DNA walker interacts with receptor‐anchored footholds, promoting receptor dimerization and transmembrane signaling amplification. Monte Carlo simulations revealed that the synchronized motion of ≈1.9×10⁴ DNA nanorobots per cell, each traveling an average of 95±15 nm, induced a net cellular migration of 87 µm, demonstrating a direct functional link between molecular‐scale interactions and macroscopic cell movement. To further refine its applications, we incorporated DNAzyme‐based modules, allowing bio‐orthogonal, stimuli‐responsive control across heterogeneous cell populations, enhancing its potential in tissue engineering and precision therapeutics.

In parallel, Liu et al. introduced a reconfigurable DNA nanobot leveraging reversible strand displacement reactions for highly controlled receptor modulation (Figure 12b).^[^ ^140^ ^]^ This system integrates aptamer‐functionalized hairpin structures that mediate controlled Met receptor dimerization and de‐dimerization, allowing reversible and dynamic regulation of cell migration. By switching between active and inactive receptor states, this DNA nanorobotic framework establishes a versatile approach for adaptive receptor tuning, offering a robust platform for synthetic cell engineering and targeted therapeutic interventions.

The emergence of DNA nanorobots as programmable, receptor‐interacting systems signifies a major breakthrough in molecular control of cellular function. As next‐generation DNA nanorobotic platforms continue to evolve, their potential to drive innovation in regenerative medicine, immunotherapy, and synthetic biology will be further unlocked, enabling precise manipulation of cellular behavior in ways previously unattainable. While DNA nanorobots hold immense promise, their translation to clinical use is hindered by critical challenges. These include immune clearance, where the body's immune system may recognize and eliminate the nanorobots, as well as limited tissue penetration, which restricts their ability to reach target cells within tissues. Additionally, large‐scale manufacturing presents difficulties due to the complexity and cost of producing these intricate structures with the required precision and consistency for widespread application.^[^ ^139^ ^]^

## 5 Conclusions and Perspectives

Advancing receptor engineering requires innovative strategies that balance precision, adaptability, and controllability to effectively regulate cellular communication and develop next‐generation biomedical applications. This review has highlighted two complementary DNA‐based engineering strategies that leverage the diverse properties of DNA molecules for receptor reprogramming: genetic and non‐genetic approaches. Genetic approaches, exemplified by various synthetic receptors, e.g. CAR‐T, SynNotch, and RASSL, utilize the coding capacity of DNA to ensure long‐term and stable receptor expression, making them ideal for applications requiring persistent cellular reprogramming (**Table** **1**).^[^ ^61^ ^]^ However, these methods rely on transcriptional and translational regulation, presenting challenges such as insertional mutagenesis risks, immunogenicity, and difficulty in controlling receptor activity and expression levels.^[^ ^15^ ^]^ Additionally, the adaptability of natural genetic components is limited, making it difficult to design synthetic receptors with highly tunable activation mechanisms. While *de novo* protein design offers potential to address these constraints, it remains an evolving field requiring further optimization.^[^ ^141^ ^]^

**Table 1:** Comparative Analysis of DNA‐based Genetic and Non‐genetic Engineering Strategies for Receptor Engineering.

Evaluation Dimension	DNA‐based Genetic Engineering	DNA‐based Non‐Genetic Engineering
Engineering Principles	Receptor function is engineered by altering the receptor‐encoding gene sequence through genetic engineering methods including domain fusion and site‐directed mutagenesis.	Receptor function is engineered by functionalized versatile DNA‐based platforms without genetic modifications.
Tool Types	Domain fusion; Site‐directed mutagenesis.	FNAs; DNA nanostructures; Dynamic DNA reactions
Regulatory Mechanism	Indirect regulation by altering the receptor‐encoding gene sequence, which determines the amino acid sequence and thus the structure and function of the receptor protein.	Direct regulation by acting as artificial ligands, structural scaffolds, or dynamic regulators that directly interact with receptors and affect receptor clustering
Application Advantages	1. Ensures long‐term and stable receptor expression. 2. Offers high programmability with orthogonal design capabilities. 3. Provides heritability, beneficial for engineering stable cell lines.	1. Eliminates the need for altering genetic code of the receptor. 2. Enables user‐defined responsiveness to stimuli. 3. Exhibits low immunogenicity, reducing the risk of immune responses.
Spatial and Temporal Resolution	1. Spatial resolution is contingent on targeted delivery methods; 2. Temporal resolution is influenced by the duration of receptor synthesis processes, typically on the scale of hours.	1. Nanoscale spatial precision for receptor clustering. 2. Dynamic responses ranging from milliseconds to minutes, facilitated by mechanisms including DNAzyme‐based cleavage and dynamic DNA reactions.
Technological Innovations	1. Domain fusion for constructing synthetic receptors with novel responsiveness and functions. 2. Receptor mutagenesis for optimizing and tuning receptor properties.	1. Dynamic control over receptor activity in response to user‐defined stimuli. 2. Enabling fine‐tuning of receptor signaling. 3. Programmable self‐assembly of DNA nanostructures for spatial organization of receptors at the nanoscale.
Clinical Translation Challenges	1. Potential risks associated with gene delivery, such as insertional mutagenesis and off‐target effects. 2. Possible immunogenic responses to engineered receptors or viral vectors. 3. Challenges in achieving targeted and sustained control over receptor expression levels in vivo.	1. Limited stability in physiological conditions due to susceptibility to nuclease degradation. 2. Challenges in achieving efficient delivery to target cells and tissues. 3. Potential interference from the complex in vivo environment, such as nonspecific binding of proteins and ions.

In contrast, DNA nanotechnology offers a highly flexible, non‐genetic alternative for receptor modulation, enabling reversible, immediate, and stimulus‐responsive control without the need for complex genetic modifications or ex vivo cell reprogramming before therapeutic applications (Table 1).^[^ ^142^ ^]^ For instance, in ex vivo expanded CAR‐T therapy, CAR‐T cell infusion is performed to administer modified T cells back into the patient. Unlike genetic approaches, the off‐the‐shelf nature of DNA‐based strategies allows the rapid assembly of receptor modulators using pre‐synthesized DNA molecular scaffolds, enabling plug‐and‐play functionality by directly functionalizing receptors of living cells.^[^ ^143^ ^]^

Furthermore, non‐genetic approaches offer unique advantages in terms of programmability. Unlike genetically encoded synthetic receptors, which require extensive design to establish *de novo* recognition properties, FNAs (aptamers and DNAzymes) can be readily selected and characterized to endow receptors with user‐defined responsiveness to diverse environmental cues.^[^ ^144^ ^]^ The responsiveness of these FNA‐engineered receptors can be finely tuned by adjusting Watson‐Crick base pairing, GC content, and secondary structures (e.g., hairpins, i‐motifs).^[^ ^90^, ^134^ ^]^ Moreover, modular DNA‐scaffolded ligands, including bivalent, polyvalent, and origami‐based addressable nanostructures, allow for highly controlled receptor clustering and stoichiometric control at the nanoscale.^[^ ^145^ ^]^ Importantly, dynamic DNA reaction networks introduce programmable and intelligent control over receptor activation. Strand displacement reactions facilitate the construction of distinctive DNA logic circuits (e.g., AND, NIMPLY), enabling highly specific, logic‐based receptor control.^[^ ^54^ ^]^ Recent advances in DNA nanorobots further expand the capabilities of dynamic receptor modulation, integrating nanoscale molecular motions with microscale cellular responses.^[^ ^55^ ^]^ Despite these advances in the field of DNA nanotechnology, practical hurdles remain for clinical translation. Specifically, enhancing the in vivo stability of DNA nanodevices, refining strategies for cell‐specific targeting and delivery, and addressing the potential for immune recognition and activation will be critical to unlocking the therapeutic potential of this technology.

Looking ahead, integrating the stability of genetic receptor engineering with the dynamic programmability of DNA nanotechnology offers a pathway to multi‐input, autonomous, and adaptive receptor regulation systems. A promising direction is the development of semisynthetic protein‐DNA chimera receptors, which link programmable extracellular dynamic DNA reactions with orthogonal intracellular signaling pathways. Furthermore, the development of DNA‐programmed membrane systems and artificial receptor modules signifies a transformative advancement in synthetic biology, enabling precise, non‐genetic control over cellular interactions and signaling pathways through programmable DNA nanostructures and logic‐driven circuits, thereby enhancing targeted therapies and immune cell efficacy.^[^ ^146^ ^]^ Beyond nanoscale receptor modulation, DNA self‐assembly is also enabling the construction of programmable artificial cells with stimulus‐responsive behaviors.^[^ ^147^ ^]^ We recently developed responsive DNA artificial cells to regulate mammalian cells via synthetic contact‐dependent communication, orthogonally regulating cellular signaling in multicellular communities and demonstrating in vivo therapeutic efficacy in muscle regeneration.^[^ ^148^ ^]^ As these technologies converge, this hybrid strategy—merging receptor engineering, programmable DNA circuits, and artificial cell assembly—is poised to redefine receptor‐controlled biological processes. These advances hold the potential to drive next‐generation smart cell therapies and programmable biological systems, transforming synthetic biology, immunotherapy, and regenerative medicine.

## Conflict of Interest

The authors declare no conflict of interest.
